# Role of Low-Affinity Calcium System Member *Fig1* Homologous Proteins in Conidiation and Trap-Formation of Nematode-trapping Fungus *Arthrobotrys oligospora*

**DOI:** 10.1038/s41598-019-40493-x

**Published:** 2019-03-14

**Authors:** Weiwei Zhang, Chengcheng Hu, Muzammil Hussain, Jiezuo Chen, Meichun Xiang, Xingzhong Liu

**Affiliations:** 1 State Key Laboratory of Mycology, Institute of Microbiology, Chinese Academy of Sciences, No. 3 Park 1, Beichen West Rd., Chaoyang District, Beijing, 100101 China; 2 University of Chinese Academy of Sciences, Beijing, 100049 China

## Abstract

*Arthrobotrys oligospora* is a typical nematode-trapping fungus capturing free-living nematodes by adhesive networks. Component of the low-affinity calcium uptake system (LACS) has been documented to involve in growth and sexual development of filamentous fungi. Bioassay showed incapacity of trap formation in *A*. *oligospora* on Water Agar plate containing 1 mM ethylene glycol tetraacetic acid (EGTA) due to Ca^2+^ absorbing block. The functions of homologous proteins (*AoFIG_1* and *AoFIG_2*) of LACS were examined on conidiation and trap formation of *A*. *oligospora*. Compared with wild type, *ΔAoFIG_1* (AOL_s00007g566) resulted in 90% of trap reduction, while *ΔAoFIG_2* (AOL_s00004g576) reduced vegetative growth rate up to 44% and had no trap and conidia formed. The results suggest that LACS transmembrane protein *fig1* homologs play vital roles in the trap-formation and is involved in conidiation and mycelium growth of *A*. *oligospora*. Our findings expand *fig1* role to include development of complex trap device and conidiation.

## Introduction

**Figure 1:**
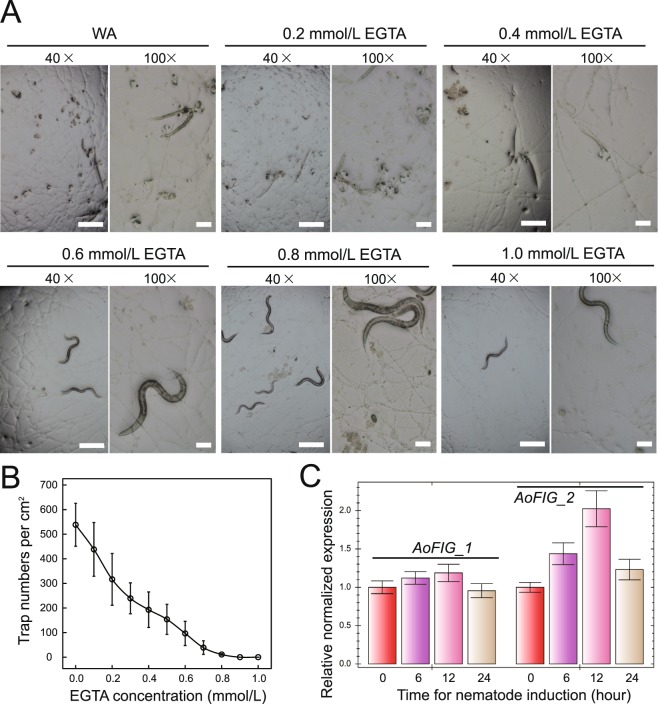
Trap-formation of *A*. *oligospora* wild type strain on Water Agar (WA) medium and WA medium containing EGTA. (**A**) Trap-formation of *A*. *oligospora* on WA medium containing different EGTA concentration. (**B**) Dosage response curve of trap numbers and different EGTA concentration. (**C**) Expression pattern of *AoFIG_1* and *AoFIG_2* during trap-formation. Bar 100× = 50 μm; Bar 40× = 200 μm.

Calcium-mediated signaling pathways are ubiquitous in various cellular processes of eukaryotic cells by regulating the level of cytosolic calcium ion^1,2^. Two major calcium uptake pathways have been identified and characterized in fungi, including the high-affinity calcium uptake system (HACS), which is activated during low calcium availability, and the low-affinity calcium uptake system (LACS), which is activated when calcium availability is high^3,4^. To date, *fig1* is the only characterized member of the LACS in fungi, with four putative trans-membrane domains and a conserved claudin motif [GɸɸGXC(n)C, where ɸ is a hydrophobic amino acid and n is any number of amino acids]^5,6^. The *fig1* is described as mating factor-induced gene 1 in *Saccharomyces cerevisiae*, because it functions in the mating pheromone and the deletion of *fig1* results in incomplete tip fusions^7^. However, in filamentous fungi, unlike in *S*. *cerevisiae*, *fig1* is more likely involved in vegetative growth, sexual and asexual development. For instance, the deletion of *fig1* resulted in slow growth and absence of mature perithecia in *Fusarium graminearum*^8^, incapacity of fruiting body development in *Neurospora crassa*^8^, and retardant mycelia growth and sharp sporulation reduction in *Aspergillus nidulans*^9^. In addition, *fig1* was associated with the vegetative growth and thigmotropism in *Candida albicans*^10^. Overall, the known functions of *fig1* in filamentous fungi are involved in the development of sexual and asexual reproductive structures.

*Arthrobotrys oligospora* is a typical nematode-trapping fungus that can capture free-living nematodes by the specialized mycelia adhesive networks^11,12^. It is a cosmopolitan species with fast vegetative growth and efficient conidia production^13,14^. However, the development of the trapping devices is still poorly understood. Bioassay showed that traps were poorly induced by free-living nematodes on the medium without Ca^2+^, suggesting that the Ca^2+^ signaling pathway is involved in the trap formation. In this study, the roles of *fig1* homologous proteins on the vegetative growth, conidiation and trap-formation of *A*. *oligospora* were investigated by gene disruption.

## Results

### Ca^2+^ is required for trap-formation

To investigate how Ca^2+^ influences trap-formation in *A*. *oligospora* induced by nematodes, the ethylene glycol tetraacetic acid (EGTA) was supplemented into the Water Agar (WA) medium to block Ca^2+^ absorption at 0.1 to 1.0 mmol/L (mM)^15^. *A*. *oligospora* was cultured on plates of WA medium and WA containing EGTA for 72 hours, and then nematodes were inoculated for trap inducing. After 48 hours, there were ca. 500 traps formed per cm^2^ on WA medium. However, only ca. 200 traps per cm^2^ were formed on the agar plate containing 0.5 mM EGTA, and no traps formed when EGTA concentration was 1 mM (Fig. 1A,B; Table S1). The results demonstrated that Ca^2+^ influx is required for trap formation.

### Screening and identification of *Fig1* domain through genome sequences

**Figure 2:**
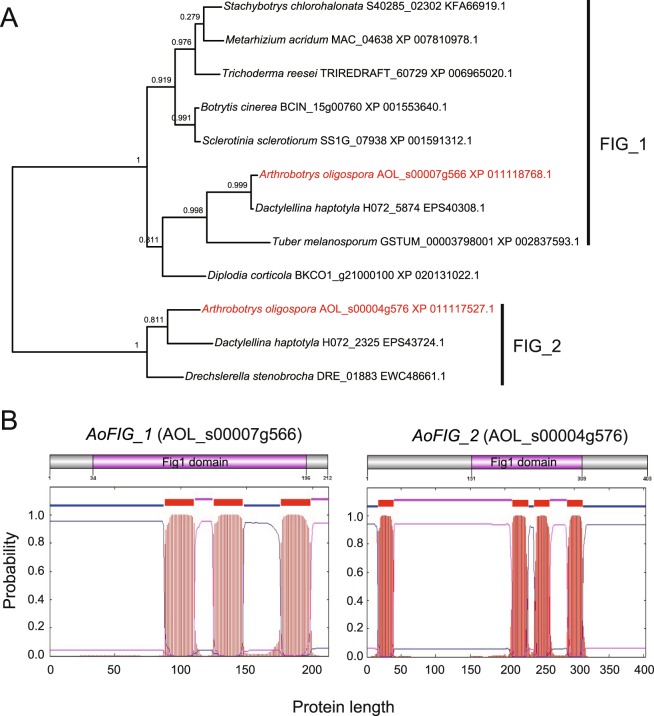
Phylogenetic analysis and protein structures prediction. (**A**) Phylogenetic relationships of the orthologs of *AoFIG_1* and *AoFIG_2*. Unrooted tree was constructed using FastTree based on protein sequences. (**B**) Putative trans-membrane regions predicted in *AoFIG_1* and *AoFIG_2* by TMHMM.

Two proteins containing Fig1 domain were identified in the published genome of *A*. *oligospora* (GenBank no. EGX4402) by HMM scanning^16,17^. AOL_s00007g566 (*AoFIG_1*) ORF sequence was 1,379 bp coding for a 212-amino-acid protein. AOL_s00004g576 (*AoFIG_2*) ORF sequence was 1,275 bp coding for a 403-amino-acid protein. Homologs of *AoFIG_1* and *AoFIG_2* were identified by blasting against NCBI database. Both the Fig1 domain appended proteins were expressed during the trap-formation detected by real-time PCR. In addition, expression *AoFIG_2* was up-regulated during the trap-formation by 2 fold change after 12-hour-induction of nematode compared with that of hyphae without nematode induction (Fig. 1C; Table S2). The most closely related protein of *AoFIG_1* was H072_5874, identified from another nematode-trapping fungus *Dactylelina haptotyla* and its homologs have been found in several phytopathogenic fungi. However, *AoFIG_2* homologs were only identified in the other two nematode-trapping fungi *D*. *haptotyla* and *Drechslerella stenobrocha*, indicating that *AoFIG_2* was conserved only in nematode-trapping fungi (Fig. 2A). Protein structure analysis by TMHMM-scan showed that only three trans-membrane regions presented in each Fig1 domain of *AoFIG_1* and *AoFIG_2* (Fig. 2B)^18^. Meanwhile, the absence of signal-P (secreting peptide signal) in both proteins indicated their location at cell membrane, which is consistent with that in the yeast^4^.

### Targeted gene disruption

**Figure 3:**
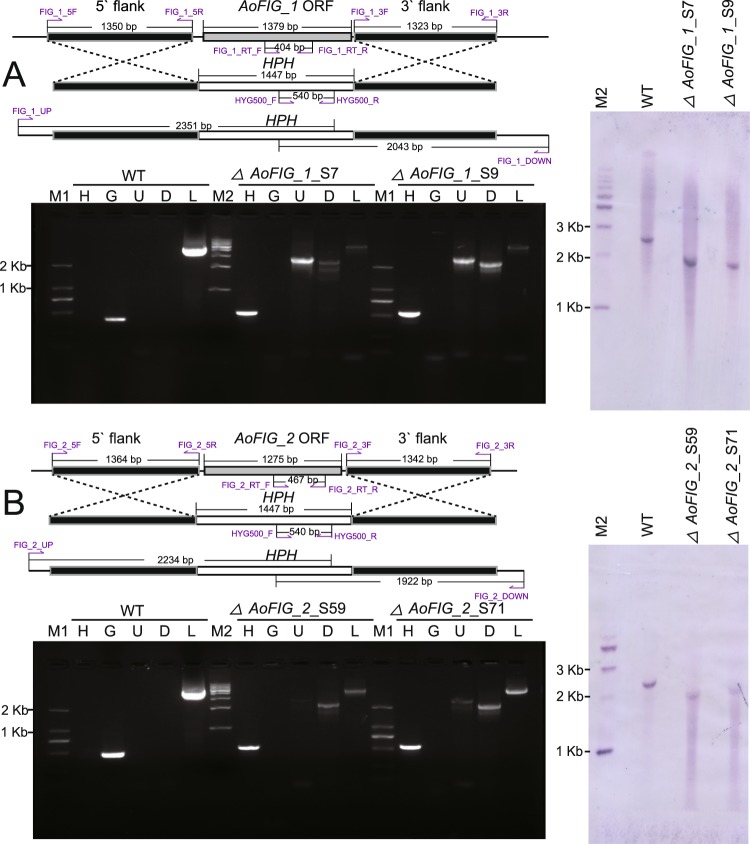
Gene disruption strategy and verification by PCR and Southern blot. (**A**) Disruption strategy and verification of *AoFIG_1* (**B**) Disruption strategy and verification of *AoFIG_2*. WT: the wild-type strain. In PCR verification, M1: DNA marker D2000; M2: DNA marker 1Kb (1 Kb and 2 Kb were marked on the right side respectively); H: hygromycin resistance gene partial sequence which was absent in WT and present in the mutants; G: gene ORF partial sequence which was absent in mutants and present in WT; U: PCR was used to ensure the correct recombination using primers UP and HYG500_R; D: PCR results using primers DOWN and HYG500_F; L: PCR results using primers DOWN and UP. The location of primers and expected length of PCR products were marked on the figure.

The functions of *AoFIG_1* and *AoFIG_2* were investigated using the strain of *A*. *oligospora* (CBS115.81) by performing targeted gene disruptions. Successful amplification of the partial transcriptional regions confirmed the presence of the two genes in this strain (Fig. 3; full gel images were shown in Figs S1 and S2). Two *AoFIG_1* disrupted mutants (*ΔAoFIG_1_*S7 and *ΔAoFIG_1_*S9) were screened from 22 transformants by PEG-mediated protoplast transformation and subsequently confirmed by PCR. Similarly, two *AoFIG_2* disrupted mutants (*ΔAoFIG_2_*S59 and *ΔAoFIG_2_*S71) were screened from 94 transformants. All the four successful knock-out mutants were then confirmed by Southern blot comparing with the wild type strain to check the single integration (Fig. 3; Southern blot strategies, full gel, and membrane images were shown in Figs S3–6). All the mutants were serially transferred onto Potato Dextrose Agar (PDA) plate containing 200 μg/mL hygromycin for five times and continuously kept on PDA plate to obtain stable mutants^19^. Considering the same phenotypes of both mutants in each gene respectively (Tables S3–5), we randomly selected *ΔAoFIG_1_*S9 and *ΔAoFIG_2_*S71 for the following phenotypic description.

### Effect of *ΔAoFIG_1* and *ΔAoFIG_2* on the growth rate and conidiation

**Figure 4:**
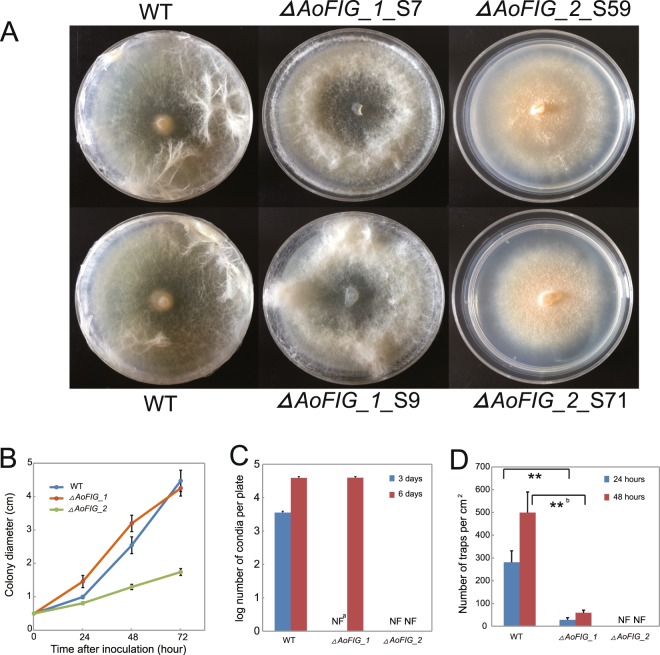
*A*. *oligospora* bio-assays. (**A**) The colony morphology of *A*. *oligospora* wild type strain (WT), *ΔAoFIG_1* and *ΔAoFIG_2* on PDA medium. (**B**) Vegetative growth rates of *ΔAoFIG_1* and *ΔAoFIG_2* on PDA medium; colony diameters were measured every 24-hour cultivation. (**C**) Capacity of conidiation on PDA medium after 3- and 6-day cultivation. (**D**) Capacity of trap-formation after induced by nematodes for 24 and 48 hours. ^a^Not found. ^b^*p* < 0.01, *t*-test.

The deletion of *ΔAoFIG_1* did not significantly affect the mycelial growth and the conidiation after six days incubation on PDA plate (Fig. 4A,B; Tables S3 and S4). However, conidiation of *ΔAoFIG_1* was not detected after three days incubation compared with wild type which produced ca. 3,500 conidia in average of each colony after 3 days incubation (Fig. 4B), indicating that disruption of *AoFIG_1* gene delayed the conidiation. The disruption of *AoFIG_2* significantly decreased the growth rate and mycelial abundance and resulted in totally loss of conidiation even after 6 days inoculation compared to wild type on PDA plates (Fig. 4B; Table S4). Overall, both Fig1 domain containing proteins of *A*. *oligospora* are involved in the conidiation but *AoFIG_2* has more crucial roles than *AoFIG_1*.

### Effect of *ΔAoFIG_1* and *ΔAoFIG_2* on trap-formation

**Figure 5:**
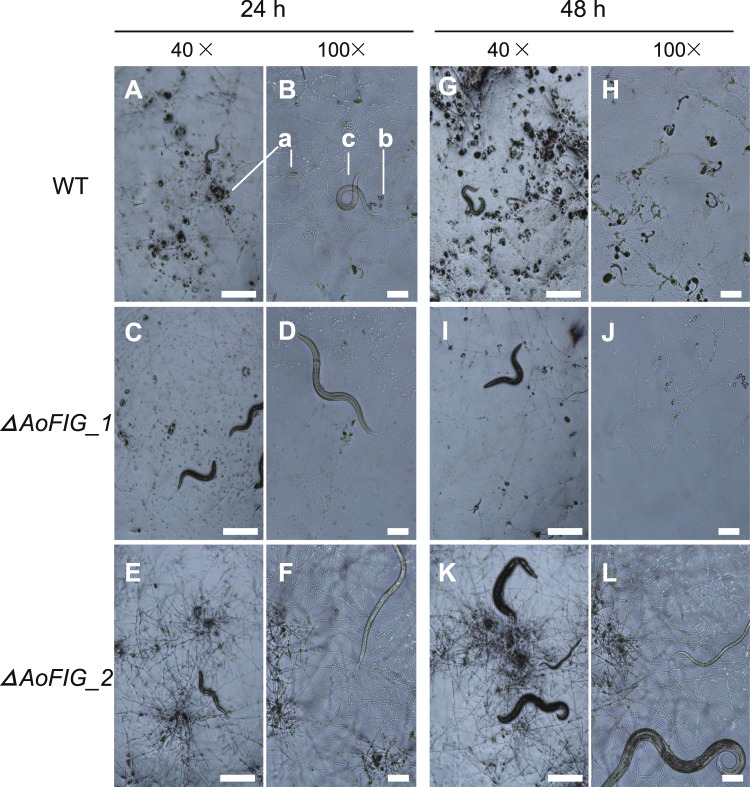
Trap-formation of *A*. *oligospora* wild-type strain (WT), *ΔAoFIG_1* and *ΔAoFIG_2*. Traps were induced by nematodes for 24 and 48 hours. The morphological characteristics of the traps were shown under both 40× and 100× magnification. a: adhesive networks; b: conidia; c: nematodes. Bar A, C, E, G, I, K = 200 μm; Bar B, D, F, H, J, L = 50 μm.

The wild-type strain could form ca. 300 and 500 trap devices per cm^2^ after 24- and 48-hour induction by nematodes on WA plates (Figs 5 and 4C; Table S5). The *ΔAoFIG_1* could form only ca. 29 (*t*-test, *p* = 2.4e^−5^, two-tailed, n = 5) and 53 (*t*-test, *p* = 1.2e^−7^, two-tailed, n = 5) traps per cm^2^ under the same condition of wild type, indicating that loss of *AoFIG_1* extremely decreased trap-formation capacity (Table S5). Whereas, there was no trap-formation after nematode induction for 48 hours, and no traps were found even after 4-day induction when *AoFIG_2* was deleted (Fig. 5). The critical roles of Fig1 domain containing proteins on the trap formation demonstrated that Ca^2+^ signaling pathway is essential for trap formation of nematode-trapping fungi.

### Roles of Fig1 domain appended proteins on abiotic stress

**Figure 6:**
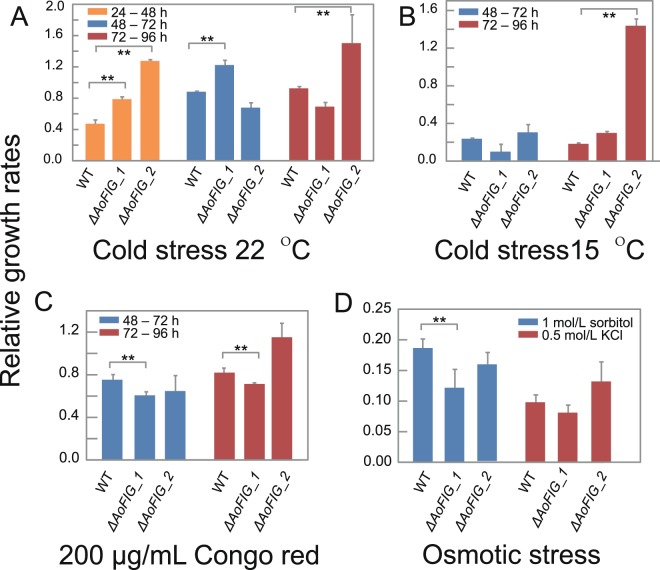
Relative growth rates of *A*. *oligospora* wild type strain (WT), disruption mutants of *ΔAoFIG_1* and *ΔAoFIG_2* to abiotic stresses. Growth rates were calculated from 24-hour to 96-hour cultivation; the growth rates on PDA at 28 °C were used as control. (**A**) Cold stress at 22 °C. (**B**) Cold stress at 15 °C. (**C**) PDA containing 200 μg/mL Congo red at 28 °C. (**D**) Osmotic stress: PDA containing 1 mol/L sorbitol and PDA containing 0.5 mol/L KCl at 28 °C.

Cold stresses were treated on PDA plates under 22 °C and 15 °C, using those under 28 °C as control. Wild-type strain was more sensitive to cold stress than *ΔAoFIG_1* during 24- to 72-hour growth (24–48 hours: *t*-test, *p* = 1.3e^−6^, two-tailed, n = 5; 48–72 hours: *t*-test, *p* = 8.1e^−7^, two-tailed, n = 5; Fig. 6A; raw data were shown in Table S6–10; phenotypes were shown in Fig. S10). The 1.5 times relative growth rates of *ΔAoFIG_2* at 15 °C than that under 28 °C showed the highest resistance to cold stress (72–96 hours: *t*-test, *p* = 2.1e^−10^, two-tailed, n = 5; Fig. 6B). *ΔAoFIG_1* produced less mycelium on PDA plates containing 200 μg/L Congo red at 28 °C with lower growth rates compared with control (48–72 hours: *t*-test, *p* = 5.5e^−4^, two-tailed, n = 5; 72–96 hours: *t*-test, *p* = 6.4e^−4^, two-tailed, n = 5; Fig. 6C), while *ΔAoFIG_2* on PDA with Congo red showed wild-type sensitivity. In addition, *ΔAoFIG_1* was hypersensitive to osmotic stress on PDA plates containing 1 mol/L sorbitol compared with the wild type strain (*t*-test, *p* = 0.003, two-tailed, n = 5).

## Discussion

Nematode-trapping fungi are a unique group that can capture and digest nematodes by means of specialized trapping structures to regulate nematode dynamics in nature^13^. Genome analysis of *A*. *oligospora* showed that MAPK signaling pathway, cell wall synthesis, energy utilization, cell division, and peroxisome are the genetic potentials for trap-formation^15^. Trap-formation has been documented to be affected by nutrient conditions using the nitrate assimilation pathway^19^ and adhesive protein *AoMad1* and MAP kinase *AoSlt2* in *A*. *oligospora*^20,21^. However, comprehensive understanding the mechanism of trap formation still remains unclear. This study characterized Fig1 domain appended proteins in *A*. *oligospora* based on genomic data^15^ and illustrated their critical roles in vegetative growth, conidiation, and trap-formation.

*Fig1* is a member of a fungus-specific protein family involved in LACS pathway and has been well documented to be involved in vegetative growth, sexual and asexual development in filamentous fungi^3–5,7,8^. Only one homolog of *fig1* was identified in other fungi, while two homologs and three trans-membrane regions in the *fig1* domains were identified in trapping fungus *A*. *oligospora* in this study. More homologs and less trans-membrane regions in *Fig1* may extend *Fig1* unique functions in nematode-trapping fungi. Those functions include cell-fusing during the trap formation and the lifestyle switch of nematode-trapping fungi from saprophytism to predatory. This expansion of calcium signaling components, in combination with the specialized roles on trap-formation, could lead to the quick cell morphology in response to the presence of nematodes.

*Fig1* is a member of the PMP22_Claudin superfamily, which is involved in membrane-to-membrane interactions and diffusion barriers formation^22^. The known functions of *fig1* in fungi, including cell-to-cell interactions and ion flux, consist well with the functions of proteins in the PMP22_Claudin superfamily in other organisms. Cell-to-cell interaction and fusion appear to be central to the function of *fig1* in yeast mating^5^. The sexual stage of *A*. *oligospora* has not been induced under laboratory conditions. However, trap formation involves in typical cellular fusion with complicated intercellular interaction^23^. *ΔAoFIG_1* decreased the efficiency of trap-formation and *ΔAoFIG_2* disabled the trap-formation, indicating that the mechanism of *fig1* on trap-formation may be resulted from the disrupted function of cell fusion. Our results illustrated that both *fig1* homologs in *A*. *oligospora* functioned in the trap-formation, and *AoFIG_2* is essential.

The loss of *fig1* did not affect the yeast cell fission of *S*. *cerevisiae* or *C*. *albicans*. However, mycelial growth was affected in certain degrees for filamentous fungi, such as few aerial hyphae development in *F*. *graminearum*^3,5,8^. Diffferent to *ΔAoFIG_1*, *ΔAoFIG_2* decreased the vegetative growth and disabled the conidiation, like that in *F*. *graminearum*, indicating that *AoFIG_2* in *A*. *oligospora* have further functions required to vegetative growth.

Mitogen-activated protein kinase (MAPK) is a cytoplasmic kinase mediating a network of interacting proteins that regulate a number of cellular processes^24^. The MAPK signaling pathway was involved in regulating the trap-formation evidenced by translational level and gene disruption^15,25^. The result of Ca^2+^ being involved in the trap-formation of *A*. *oligospora* in this study might be through MAPK pathway because Ca^2+^ is a crucial signal ion in MAPK pathway. Furthermore, the *ΔAoFIG_2* showed the inability of conidiation and trap-formation, suggesting that both *fig1* homologs might share the same Ca^2+^ signaling pathway regulation. Blocking the Ca^2+^ absorption by EGTA supported the function of Ca^2+^ signaling pathway in the trap-formation.

Soil salinity is one of the most significant abiotic stresses. Combating to osmotic stress turns to be crucial for nematode-trapping fungi to survive in the soil. Ca^2+^ is involved in the translocation of membrane-binding proteins involved in osmotic resistance^26^. Sensitivity to osmotic stress by disruptions of *fig1* containing proteins might result from the disturbed protein translocation. At the same time, the proteins involved in Ca^2+^ mediated translocation can also play roles in signal transduction and cell morphology^26,27^. Overall, these also might be the important factors blocking the trap-formation by the disruption of *AoFIG_2*.

## Materials and Methods

### Strains and growth conditions

The wild-type strain of *A*. *oligospora* (CBS115.81) was purchased from CBS. It was maintained on Corn Meal Agar (CMA) medium and Potato Dextrose Agar (PDA) medium (BD^TM^) at 26 °C. *Caenorhabditis elegans* was grown in NGM liquid medium and fed with *Escherichia coli* stain OP50 at 23 °C, 200 rpm^28^.

### Induction of traps on medium containing different concentrations of EGTA

To characterize the influence of Ca^2+^ on trap-formation, the wild-type stain of *A*. *oligospora* was cultivated for three days on WA and WA containing EGTA with the following concentrations: 0.1 to 1 mM^15^. Traps were induced and calculated by adding about 1,000 nematodes per plate for 48 hours.

### Real-time PCR of *AoFIG_1* and *AoFIG_2*

Conidia of the wild-type stain of *A*. *oligospora* were cultivated for three days on WA plates covered by cellophane. Mycelia were harvested after induction of about 1,000 nematodes for 0, 6, 12, 24 hours. RNA was extracted by TRIzol. The cDNA was obtained by reverse transcription kits (TIANGEN, Beijing China). Real-time PCR was performed using SYBR Green real-time PCR Master mix (TOYOBO, Osaka Japan).

### Gene knockout of *AoFIG_1* and *AoFIG_2*

The genes coding for protein *AoFIG_1* (AOL_s00007g566 GenBank no. XP 011118768.1) and *AoFIG_2* (AOL_s00004g576 GenBank no. XP 011117527.1) were knocked out by homologous recombination^25^. Primers were shown in Table S11. The 5′ and 3′ flanking sequences were amplified with the primers 5F and 5R and 3F and 3R, respectively (Fig. S7). The hygromycin resistant gene cassette was amplified with the primers HYG_F and HYG_R from the vector pCT74, containing P-trpC promoter and hygromycin resistant gene, without terminator (Fig. S8)^29^. The knockout cassettes were constructed using double-joint PCR for each gene (Fig. S9)^30^. PCR products were purified by Tiangen^TM^ PCR products purify kits. Then the protoplasts of *A*. *oligospora* were transformed by PEG mediated transformation^31^. Transformants were selected by PDA medium (BD^TM^) containing 200 μg/mL hygromycin and were verified by PCR using primers HYG500_F and HYG500_R^19^. Positive transformants were then confirmed by PCR with the negative result using primers RT_F and RT_R. Successful homologous recombinations were verified by the positive PCR results using the primers UP and HYG500_R and DOWN and HYG500_F, respectively (Table S11, Fig. 2).

### Southern blot

For Southern blot, DNA sequences of *AoFIG_1* and *AoFIG_2* including the up- and down-streams in strain CBS115.81 were sequenced to confirm the restriction endonucleases (DNA sequences were shown in dataset 1 and dataset 2). The wild type and mutants were cultivated on PDA plates for 7 days. The 5′ flanking sequences of both *AoFIG_1* and *AoFIG_2* were used to make probes. DNA was extracted using CTAB method^32^. DNA for *AoFIG_1* and *AoFIG_2* were digested by KpnI and SalI, DraI and SalI, respectively (Figs S5 and S6). Southern blot was performed using DIG High Prime DNA Labeling and Detection Starter Kit I (Roche) according to the manufacture protocol.

### Comparison of growth rates and conidiation

To characterize the growth rates of the mutants and the wild-type strain, the wild-type strain and each mutant were cultivated by inoculating 5-mm diameter plugs from five-day-old PDA cultures onto 6-cm diameter plates containing 5 mL PDA medium without hygromycin resistance. The mycelial growth rates were compared by measuring the colony diameters every 24 hours. Spores were washed by water containing 0.1% tween 80 and harvested from the cultures as described above. The conidiation capacity was compared by calculating the total conidia produced after three- and six-day cultivation, respectively.

### Comparison of trap-formation capacity

To compare the ability of trap-formation, the wild-type strain and mutants were cultivated on 9-cm Water Agar (WA) plates by spreading approximated 0.1 g grinded hyphae (because the *ΔAoFIG_2* strains lose the capacity of sporulation). After 3-day cultivation at 28 °C, about 1,000 *C*. *elegans* in 500 μL water were added onto the center of each plate. Traps were calculated in the 4 cm^2^ region at the plate center after 24- and 48-hour induction.

### Statistical analysis

Data from experiments are expressed as mean ± SD. Data were analyzed using SPSS 11.0 software (SPSS Inc.). Significant differences were determined by values of *p* < 0.01 using *t*-test.

## Supplementary information

Supplementary information

## Data Availability

Supporting data have been included in the supplementary.

## References

[1] Berridge MJ, Bootman MD, Roderick HL (2003). Calcium signaling: dynamics, homeostasis and remodeling. Nat. Rev. Mol. Cell Biol..

[2] Patergnani S (2011). Calcium signaling around mitochondria associated membranes (MAMs). Cell Commun. Signal..

[3] Yang M (2011). *Fig1* facilitates calcium influx and localizes to membranes destined to undergo fusion during mating in *Candida albicans*. Eukaryot. Cell.

[4] Muller EM, Locke EG, Cunningham KW (2001). Differential regulation of two Ca^2+^ influx systems by pheromone signaling in *Saccharomyces cerevisiae*. Genetics.

[5] Muller EM, Mackin NA, Erdman SE, Cunningham KW (2003). Fig1p facilitates Ca^2+^ influx and cell fusion during mating of *Saccharomyces cerevisiae*. J. Biol. Chem..

[6] Van Itallie CM, Anderson JM (2006). Claudins and epithelial paracellular transport. Annu. Rev. Physiol..

[7] Aguilar PS, Engel A, Walter P (2007). The plasma membrane proteins *prm1* and *fig1* ascertain fidelity of membrane fusion during yeast mating. Mol. Biol. Cell.

[8] Cavinder B, Trail F (2012). Role of *fig1*, a component of the low-affinity calcium uptake system, in growth and sexual development of filamentous fungi. Eukaryot. Cell.

[9] Zhang, S. *et al*. *FigA*, a putative homolog of low-affinity calcium system member *fig1* in *Saccharomyces cerevisiae*, is involved in growth and asexual and sexual development in *Aspergillus nidulans*. *Eukaryot*. *Cell* **13**, 295 (2014). 10.1128/EC.00257-13 PMC3910983 24376003

[10] Alexandra B (2007). Hyphal orientation of *Candida albicansis* regulated by a calcium-dependent mechanism. Curr. Biol..

[11] Pramer D (1964). Nematode-trapping fungi. Science.

[12] Yang Y, Yang E, An Z, Liu X (2007). Evolution of nematode-trapping cells of predatory fungi of the Orbiliaceae based on evidence from rRNA-encoding DNA and multiprotein sequences. Proc. Nat. Acad. Sci. USA.

[13] Li J (2015). Molecular mechanisms of nematode-nematophagous microbe interactions: basis for biological control of plant-parasitic nematodes. Annu. Rev. Phytopath..

[14] Niu X, Zhang K (2011). *Arthrobotrys oligospora*: a model organism for understanding the interaction between fungi and nematodes. Mycology.

[15] Shaw BD, Hoch HC (2000). Ca^2+^ regulation of *Phyllosticta ampelicida* pycnidiospore germination and appressorium formation. Fungal Genet. Biol..

[16] Yang J (2011). Genomic and proteomic analyses of the fungus *Arthrobotrys oligospora* provide insights into nematode. PLoS Pathog..

[17] Finn, R. D. Pfam: the protein families database. Encyclopedia of Genetics, Genomics, Proteomics and Bioinformatics. John Wiley & Sons, Ltd. (2005).

[18] Chen Y, Yu P, Luo J, Jiang Y (2003). Secreted protein prediction system combining CJ-SPHMM, TMHMM, and PSORT. Mamm. Genome.

[19] Liang L (2016). The nitrate assimilation pathway is involved in the trap formation of *Arthrobotrys oligospora*, a nematode-trapping fungus. Fungal Genet. Biol..

[20] Liang L (2015). A proposed adhesin AoMad1 helps nematode-trapping fungus *Arthrobotrys oligospora* recognizing host signals for life-style switching. Fungal Genet. Biol..

[21] Zhen Z (2018). Map kinase Slt2 orthologs play similar roles in conidiation, trap formation, and pathogenicity in two nematode-trapping fungi. Fungal Genet. Biol..

[22] Rosenthal R (2010). Claudin-2, a component of the tight junction, forms a paracellular water channel. J. Cell Sci..

[23] Nordbring-Hertz B, Friman E, Veenhuis M (1989). Hyphal fusion during initial stages of trap formation in *Arthrobotrys oligospora*. Antonie Van Leeuwenhoek.

[24] Seger R, Krebs G (1995). The MAPK signaling cascade. FASEB J..

[25] Brenner ST (1974). Genetics of *Caenorhabditis elegans*. Genetics.

[26] Lee S (2004). Proteomic identification of annexins, calcium-dependent membrane binding proteins that mediate osmotic stress and abscisic acid signal transduction in *Arabidopsis*. Plant Cell.

[27] Connolly JH, Jellison J (1995). Calcium translocation, calcium oxalate accumulation, and hyphal sheath morphology in the white-rot fungus *Resinicium bicolor*. Can. J. Bot..

[28] Colot, H. V. *et al*. A high-throughput gene knockout procedure for *Neurospora* reveals functions for multiple transcription factors. Proc. Nat. Acad. Sci. USA **103**, 10352–7 (2006). 10.1073/pnas.0601456103 PMC1482798 16801547

[29] Andrie RM, Martinez JP, Ciuffetti LM (2005). Development of Toxa and Toxb promoter-driven fluorescent protein expression vectors for use in filamentous ascomycetes. Mycologia.

[30] Yu JH (2004). Double-joint PCR: a PCR-based molecular tool for gene manipulations in filamentous fungi. Fungal Genet. Biol..

[31] Jin X, Mo MH, Huang XW, Zhang KQ (2005). Improvement on genetic transformation in the nematode-trapping fungus *Arthrobotrys oligospora*, and its quantification on dung samples. Mycopathologia.

[32] Góesneto A, Loguercioleite C, Guerrero RT (2005). DNA extraction from frozen field-collected and dehydrated herbarium fungal basidiomata: performance of SDS and CTAB-based methods. Biotemas.

