# Supplementary material for: Role of Low-Affinity Calcium System Member Fig1 Homologous Proteins in Conidiation and Trap-Formation of Nematode-trapping Fungus Arthrobotrys oligospora
Source: Sci Rep. 2019 Mar 14;9:4440. doi: 10.1038/s41598-019-40493-x (PMC6418195; doi:10.1038/s41598-019-40493-x)
Supplement: Supplementary file 1 — Supplementary information [file 41598_2019_40493_MOESM1_ESM.pdf]

Supplementary Information

Role of Low-Affinity Calcium System Member *fig1* Homologous Proteins in Conidiation and Trap-Formation of Nematode-trapping Fungus *Arthrobotrys oligospora*

Weiwei Zhang[1,2,§], Chengcheng Hu[1,2,§], Muzammil Hussain[1,2], Jiezuo Chen[1,2],

Meichun Xiang[1], & Xingzhong Liu[1,*]

Supplementary Table S1. Induction of trap formation of *A. oligospora* on water agar medium containing EGTA. Rep: seven replicates; S.D.: Standard Deviation.

EGTA (mmol/L)	Rep1	Rep2	Rep3	Rep4	Rep5	Rep6	Rep7	Average	S.D.
0.0	441	577	616	498	645	574	418	538	87.33
0.1	561	331	292	438	569	380	497	438	109.47
0.2	312	346	276	309	430	424	118	316	105.22
0.3	214	203	355	206	301	189	207	239	63.03
0.4	225	105	212	176	314	109	209	193	72.31
0.5	128	235	69	121	132	234	158	154	61.17
0.6	119	32	132	95	157	28	113	97	49.25
0.7	32	3	71	14	39	79	34	39	27.75
0.8	8	9	12	16	9	9	14	11	3.06
0.9	0	0	0	0	0	0	0	0	0.00
1.0	0	0	0	0	0	0	0	0	0.00

Supplementary Table S2. Expression of *AoFIG_1* and *AoFIG_2* during trap-formation detected by real-time PCR.

Target	Sample	Ctrl	Expression	Expression SEM	Corrected Expression SEM	Mean Cq	Cq SEM
AoFIG_2	0h	*	1.00000	0.06428	0.06428	25.83	0.07591
AoFIG_2	12h		2.02339	0.23432	0.23432	29.48	0.11990
AoFIG_2	24h		1.23026	0.13345	0.13345	23.49	0.07863
AoFIG_2	6h		1.43663	0.14211	0.14211	27.32	0.10785
AoFIG_1	0h	*	1.00000	0.08331	0.08331	25.11	0.10774
AoFIG_1	12h		1.18671	0.11234	0.11234	29.52	0.07151
AoFIG_1	24h		0.95492	0.09094	0.09094	23.13	0.02389
AoFIG_1	6h		1.12096	0.08167	0.08167	26.96	0.04811
AoActin	0h	*				25.46	0.05328
AoActin	12h					30.12	0.11635
AoActin	24h					23.42	0.13530
AoActin	6h					27.47	0.09346

Supplementary Table S3. Conidiation (conidia numbers) of wild type (WT) strain and the mutants. Rep: five replicates; S.D.: Standard Deviation.

**Strain**	**Rep1**	**Rep2**	**Rep3**	**Rep4**	**Rep5**	**Average**	**S. D.**
**3-day-cultivation**							
WT	3900	3200	3350	3500	3950	3580	332.79
*ΔAoFIG_1*_S7	0	0	0	0	0	0	0
*ΔAoFIG_1*_S9	0	0	0	0	0	0	0
*ΔAoFIG_2*_S59	0	0	0	0	0	0	0
*ΔAoFIG_2*_S71	0	0	0	0	0	0	0
**6-day-cultivation**							
WT	38333	44000	38833	34333	40667	39233	3526.88
*ΔAoFIG_1*_S7	42500	34833	43167	39500	38000	39600	3406.69
*ΔAoFIG_1*_S9	36167	37333	39833	34833	38833	37400	2005.55
*ΔAoFIG_2*_S59	0	0	0	0	0	0	0
*ΔAoFIG_2*_S71	0	0	0	0	0	0	0

Supplementary Table S4. Growth rate (colony diameter (cm)) of wild type (WT) strain and the mutants. Rep: five replicates; S.D.: Standard Deviation.

Strain	Rep1	Rep2	Rep3	Rep4	Rep5	Average	S. D.
**24-hour-cultivation**							
WT	1.03	1.05	0.97	1.02	0.93	1.00	0.048
*ΔAoFIG_1*_S7	1.34	1.26	1.42	1.58	1.71	1.46	0.18
*ΔAoFIG_1*_S9	1.18	1.21	1.42	1.43	1.61	1.37	0.18
*ΔAoFIG_2*_S59	0.78	0.83	0.86	0.84	0.75	0.81	0.045
*ΔAoFIG_2*_S71	0.59	0.72	0.86	0.75	0.56	0.70	0.12
**48-hour-cultivation**							
WT	2.55	2.76	2.13	2.59	2.72	2.55	0.25
*ΔAoFIG_1*_S7	3.50	3.28	3.33	2.92	2.98	3.20	0.24
*ΔAoFIG_1*_S9	3.50	3.21	3.33	2.84	2.93	3.16	0.27
*ΔAoFIG_2*_S59	1.22	1.28	1.37	1.39	1.23	1.30	0.079
*ΔAoFIG_2*_S71	1.17	1.25	1.36	1.29	1.20	1.25	0.075
**72-hour-cultivation**							
WT	4.71	4.59	3.93	4.67	4.49	4.48	0.32
*ΔAoFIG_1*_S7	4.18	3.93	4.44	4.21	4.53	4.26	0.24
*ΔAoFIG_1*_S9	4.08	3.89	4.40	4.11	4.35	4.17	0.21
*ΔAoFIG_2*_S59	1.75	1.83	1.86	1.67	1.62	1.75	0.10
*ΔAoFIG_2*_S71	1.75	1.71	1.82	1.59	1.59	1.69	0.11

Supplementary Table S5. Trap-formation (trap numbers) of wild type (WT) strain and the mutants. Rep: five replicates; S.D.: Standard Deviation.

**Strain**	**Rep1**	**Rep2**	**Rep3**	**Rep4**	**Rep5**	**Average**	**S. D.**
**24-hour-induction of nematode**							
WT	319	253	237	247	350	281	50.20
*ΔAoFIG_1*_S7	21	33	30	17	41	28	9.58
*ΔAoFIG_1*_S9	19	35	25	39	26	29	8.07
*ΔAoFIG_2*_S59	0	0	0	0	0	0	0.00
*ΔAoFIG_2*_S71	0	0	0	0	0	0	0.00
**48-hour-induction of nematode**							
WT	531	448	497	388	629	499	90.62
*ΔAoFIG_1*_S7	69	53	51	47	73	59	11.61
*ΔAoFIG_1*_S9	51	63	45	66	42	53	10.69
*ΔAoFIG_2*_S59	0	0	0	0	0	0	0.00
*ΔAoFIG_2*_S71	0	0	0	0	0	0	0.00

Supplementary Table S6. Colony diameter (cm) and growth rate (24 hours to 96 hours (cm)) of wild type strain. Rep: five replicates; NG: no observed growth, colony diameter is initial 0.5 cm.

Abiotic stress	Time (hours)	Rep1	Rep2	Rep3	Rep4	Rep5	Growth rate
**28 ℃**	24	1.05	1.01	1.07	1.02	1.03	
	48	2.75	2.31	2.69	2.47	2.61	
	72	4.68	4.55	4.50	4.75	4.61	
	96	6.47	6.62	6.89	6.64	6.71	5.63 ±0.14
**22 ℃**	24	0.77	0.73	0.75	0.78	0.74	
	48	1.54	1.42	1.46	1.60	1.39	
	72	3.37	3.20	3.27	3.41	3.21	
	96	5.32	5.15	5.13	5.25	5.07	4.43 ±0.08
**15 ℃**	24	NG	NG	NG	NG	NG	
	48	0.80	0.82	0.77	0.83	0.75	
	72	1.28	1.29	1.28	1.32	1.23	
	96	1.65	1.66	1.64	1.72	1.60	1.15 ±0.04
**200 μg/mL Congo Red**	24	0.72	0.74	0.73	0.71	0.73	
	48	1.18	1.20	1.21	1.05	1.12	
	72	2.77	2.88	2.76	2.47	2.60	
	96	4.43	4.50	4.38	4.30	4.26	3.64 ±0.09
**1.0 M sorbitol**	96	1.51	1.59	1.57	1.43	1.65	1.05 ±0.08
**0.5 M KCl**	96	1.02	0.96	1.05	1.14	1.09	0.55 ±0.07

Supplementary Table S7. Colony diameter (cm) and growth rate (24 hours to 96 hours (cm)) of *ΔAoFIG_1_S*7**.** Rep: five replicates; NG: no observed growth, colony diameter is initial 0.5 cm.

**Abiotic stress**	**Time (hours)**	**Rep1**	**Rep2**	**Rep3**	**Rep4**	**Rep5**	**Growth rate**
**28 ℃**	24	1.20	1.39	1.55	1.59	1.46	
	48	2.75	3.27	3.35	3.26	3.14	
	72	4.34	4.41	4.63	4.54	4.70	
	96	5.94	6.10	6.41	6.12	6.57	4.79 ±0.21
**22 ℃**	24	0.93	1.01	1.00	0.93	1.01	
	48	2.40	2.23	2.19	2.49	2.23	
	72	4.00	3.62	3.71	3.93	3.62	
	96	4.86	4.73	4.71	4.87	4.73	3.80 ±0.12
**15 ℃**	24	NG	NG	NG	NG	NG	
	48	0.79	0.81	0.69	0.73	0.68	
	72	0.79	0.92	0.79	0.75	0.85	
	96	1.34	1.31	1.40	1.41	1.34	0.86 ±0.04
**200 µg/mL Congo Red**	24	NG	NG	NG	NG	NG	
	48	0.70	0.72	0.80	0.69	0.72	
	72	1.35	1.21	1.35	1.34	1.21	
	96	2.54	2.43	2.75	2.70	2.23	2.03 ±0.21
**1.0 M sorbitol**	96	1.09	0.94	1.12	0.92	1.16	0.54 ±0.11
**0.5 M KCl**	96	0.83	0.81	0.85	0.85	0.84	0.34 ±0.02

Supplementary Table S8. Colony diameter (cm) and growth rate (24 hours to 96 hours (cm)) of *ΔAoFIG_1*_S9**.** Rep: five replicates; NG: no observed growth, colony diameter is initial 0.5 cm.

Abiotic stress	Time (hours)	Time (hours)	Rep1	Rep2	Rep3	Rep4	Rep5	Growth rate
**28 ℃**	24	1.60	1.65	1.60	1.62	1.62	1.60	
	48	3.31	3.37	3.39	3.37	3.38	3.31	
	72	4.40	4.51	4.69	4.43	4.51	4.40	
	96	6.03	6.06	6.10	6.04	6.07	6.03	4.44 ±0.04
**22 ℃**	24	1.29	1.19	1.22	1.19	1.20	1.29	
	48	2.67	2.56	2.53	2.64	2.58	2.67	
	72	4.02	3.97	4.03	3.98	3.99	4.02	
	96	5.06	5.17	4.96	5.07	5.07	5.06	3.85 ±0.10
**15 ℃**	24	NG	NG	NG	NG	NG	NG	
	48	0.78	0.87	0.76	0.78	0.89	0.78	
	72	0.94	0.88	0.98	0.93	0.92	0.94	
	96	1.36	1.32	1.43	1.42	1.39	1.36	0.88 ±0.05
**200 μg/mL Congo red**	24	NG	NG	NG	NG	NG	NG	
	48	0.88	0.85	0.77	0.88	0.83	0.88	
	72	1.52	1.57	1.50	1.55	1.54	1.52	
	96	2.63	2.66	2.63	2.65	2.65	2.63	2.14 ±0.01
**1.0 M sorbitol**	96	1.09	0.94	1.12	0.92	1.16	1.09	0.55 ±0.11
**0.5 M KCl**	96	0.87	0.84	0.81	0.85	0.87	0.87	0.35 ±0.02

Supplementary Table S9. Colony diameter (cm) and growth rate (24 hours to 96 hours (cm)) of *ΔAoFIG_2_*S59**.** Rep: five replicates; NG: no observed growth, colony diameter is initial 0.5 cm.

Abiotic stress	Time (hours)	Time (hours)	Rep1	Rep2	Rep3	Rep4	Rep5	Growth rate
**28 ℃**	24	0.76	0.75	0.84	0.82	0.80	0.76	
	48	1.30	1.28	1.44	1.40	1.37	1.30	
	72	1.67	1.62	1.81	1.75	1.73	1.67	
	96	2.15	2.08	2.11	2.08	2.09	2.15	1.39 ±0.04
**22 ℃**	24	0.80	0.80	0.84	0.80	0.81	0.80	
	48	1.43	1.46	1.45	1.44	1.45	1.43	
	72	1.73	1.86	1.76	1.79	1.80	1.73	
	96	2.21	2.27	2.16	2.23	2.22	2.21	1.61 ±0.14
**15 ℃**	24	NG	NG	NG	NG	NG	NG	
	48	0.67	0.74	0.73	0.68	0.74	0.67	
	72	0.87	0.77	0.78	0.79	0.83	0.87	
	96	1.55	1.47	1.50	1.59	1.63	1.55	1.00 ±0.03
**200**	24	NG	NG	NG	NG	NG	NG	
**μg/mL**	48	0.74	0.71	0.69	0.70	0.70	0.74	
**Congo**	72	0.98	1.01	1.05	0.96	1.04	0.98	
**Red**	96	1.52	1.51	1.57	1.41	1.43	1.52	0.93 ±0.04
**1.0 M sorbitol**	96	0.72	0.78	0.78	0.67	0.73	0.72	0.21 ±0.03
**0.5 M KCl**	96	0.67	0.69	0.67	0.65	0.69	0.67	0.18 ±0.03

Supplementary Table S10. Colony diameter (cm) and growth rate (24 hours to 96 hours (cm)) of *ΔAoFIG_2_S*71**.** Rep: five replicates; NG: no observed growth, colony diameter is initial 0.5 cm.

**Abiotic stress**	**Time (hours)**	**Time (hours)**	**Rep1**	**Rep2**	**Rep3**	**Rep4**	**Rep5**	**Growth rate**
**28 ℃**	24	0.76	0.72	0.74	0.72	0.73	0.76	
	48	1.23	1.22	1.24	1.22	1.23	1.23	
	72	1.72	1.70	1.65	1.66	1.67	1.72	
	96	2.20	2.14	2.08	2.11	2.11	2.20	1.31 ±0.05
**22 ℃**	24	0.82	0.83	0.83	0.83	0.83	0.82	
	48	1.44	1.47	1.45	1.46	1.46	1.44	
	72	1.73	1.82	1.74	1.74	1.77	1.73	
	96	2.64	2.30	2.36	2.49	2.38	2.64	1.41 ±0.06
**15 ℃**	24	NG	NG	NG	NG	NG	NG	
	48	0.71	0.79	0.67	0.74	0.7	0.71	
	72	0.89	0.88	0.84	0.86	0.83	0.89	
	96	1.53	1.51	1.46	1.49	1.53	1.53	1.05 ±0.06
**200 µg/mL Congo Red**	24	NG	NG	NG	NG	NG	NG	
	48	0.62	0.67	0.61	0.60	0.63	0.62	
	72	0.82	1.03	0.86	0.94	0.94	0.82	
	96	1.42	1.50	1.41	1.40	1.44	1.42	0.99 ±0.07
**1.0 M sorbitol**	96	0.72	0.75	0.66	0.71	0.72	0.72	0.24 ±0.05
**0.5 M KCl**	96	0.67	0.68	0.65	0.70	0.72	0.67	0.17 ±0.02

Supplementary Table S11. Primers used in this study.

Hygromycin resistance gene	
HYG500_F	ttgcaagacctgcctgaaaccgaactgccc
HYG500_R	aaccaagctctgatagagttggtcaagacc
HYG_F	gataccgtcgacgttaactgatattgaaggagc
HYG_R	tcgaggtcgaggtcgacgttaactggttcccgg
AoFIG_1	
FIG_1_RT_F	GAAACATAGTTGCAAACGCCATGC
FIG_1_RT_R	GCTGCTAGCGGTGCTCGATCCGCTG
FIG_1_5F	TAGCGGATATCCTTCAGTACATAGAAAGG
FIG_1_5R	gctccttcaatatcagttaacgtcgacggtatcTGTCGTTATTTGCCAGTTCGTGATGAG
FIG_1_3F	ccgggaaccagttaacgtcgacctcgacctcgaCTCGCCTCCAGGGGTATAAGTCTTATTC
FIG_1_3R	GAACTTGGTCTACCTTAGCGAAAGCTTG
FIG_1_UP	GATCCCTACGCAAAATACAGTCTCAG
FIG_1_DOWN	CCTAGGGTCTTTCTCGACAAACAC
AoFIG_2	
FIG_2_5F	TTGGATTGTTACACAACGGTCAATGTG
FIG_2_5R	gctccttcaatatcagttaacgtcgacggtatcGATGGTTGGTGTTGATAGCTGGTCTAG
FIG_2_3F	ccgggaaccagttaacgtcgacctcgacctcgaGCTGAGTATGTGGGAAGAGTCTATTAGC
FIG_2_3R	GGTTTAAAAAAGCTTCGACTAGCCTTG
FIG_2_RT_F	AGGATAAGATTGCATTCTCCCTTCC
FIG_2_RT_R	GATGGTTGGTGTTGATAGCTGGTCTAG
FIG_2_UP	AATCTGATCAACCCCCAGGCTTGTG
FIG_2_DOWN	CCGATTCGCTTGTTTTAACCTTCAG
Real-time PCR	
AOActin_RT_F	GTATGATGGCCACTTTCTCAGTCG
AOActin_RT_R	GCTCCTCATCGGCATAAGCCTCC

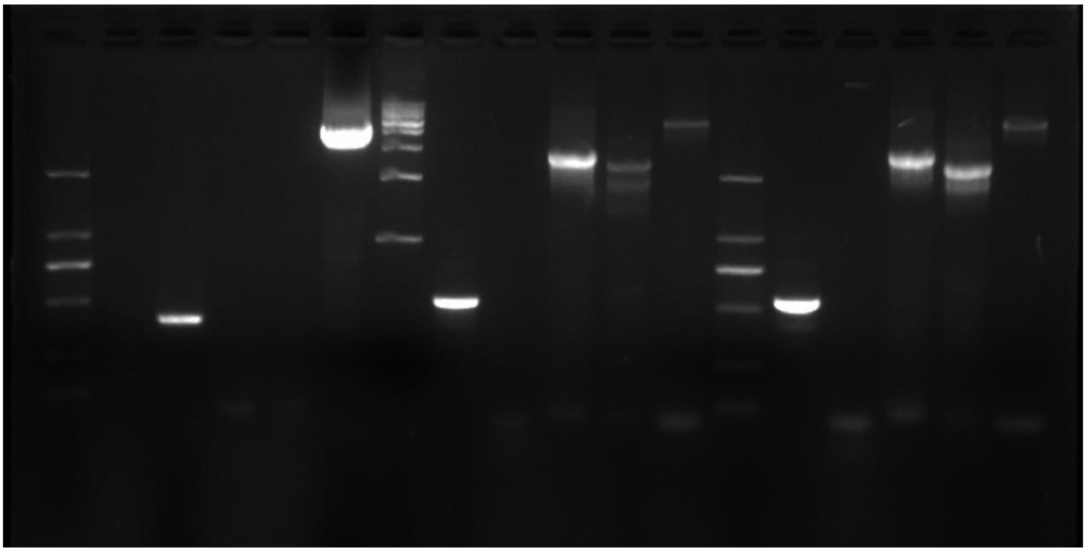

Supplementary Fig. S1. Full gel image of *AoFIG_1* mutant used in Fig. 3

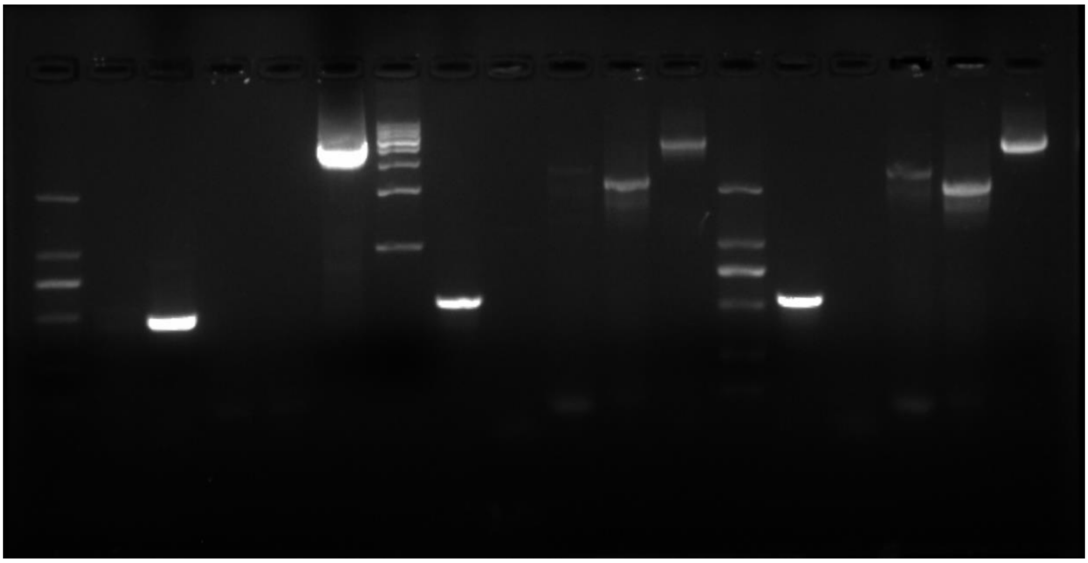

Supplementary Fig. S2. Full gel image of *AoFIG_2* mutant used in Fig. 3

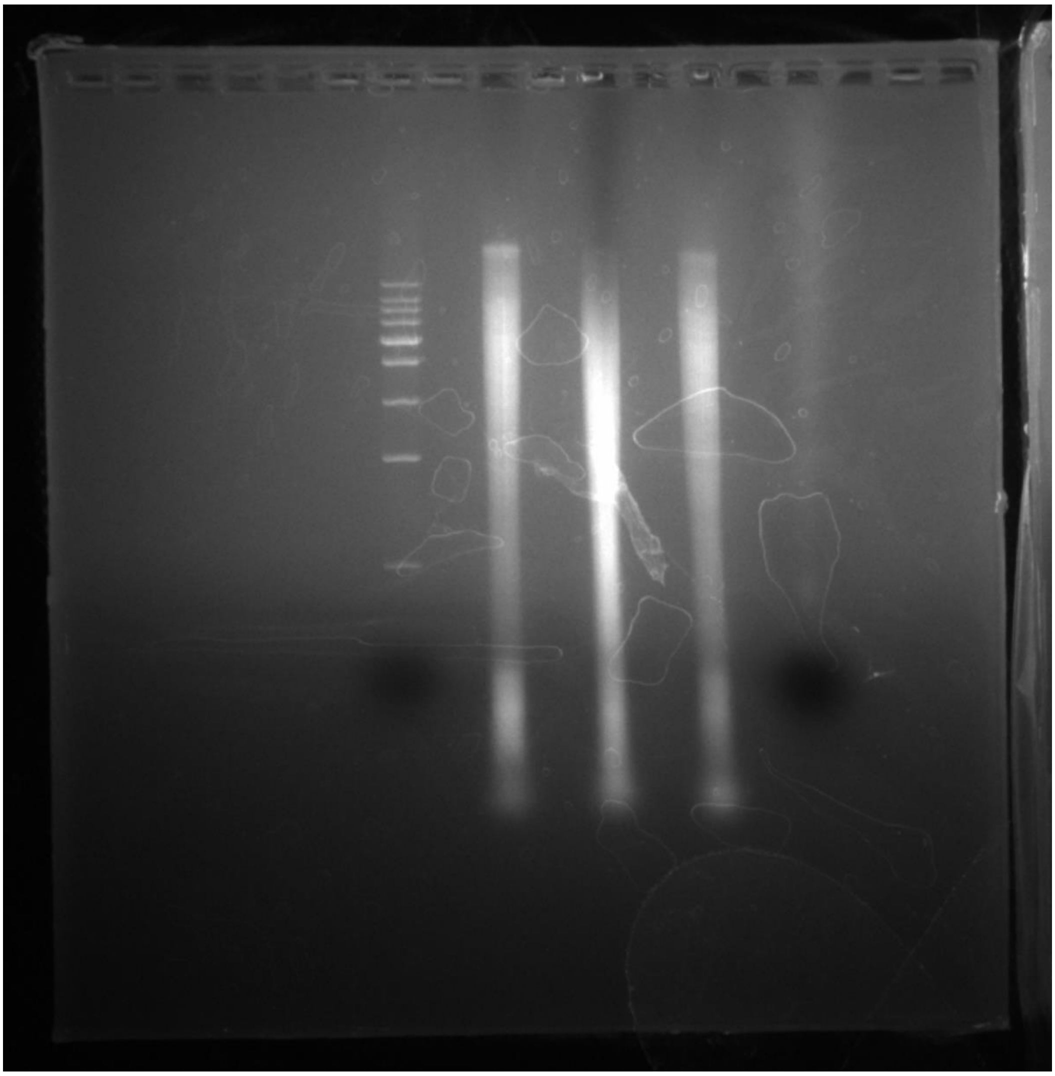

Supplementary Fig. S3. Full gel image of *AoFI1* for Southern blot. Left to right: 1Kb DNA marker; WT; *ΔAoFIG_1*_S7; *ΔAoFIG_1*_S9.

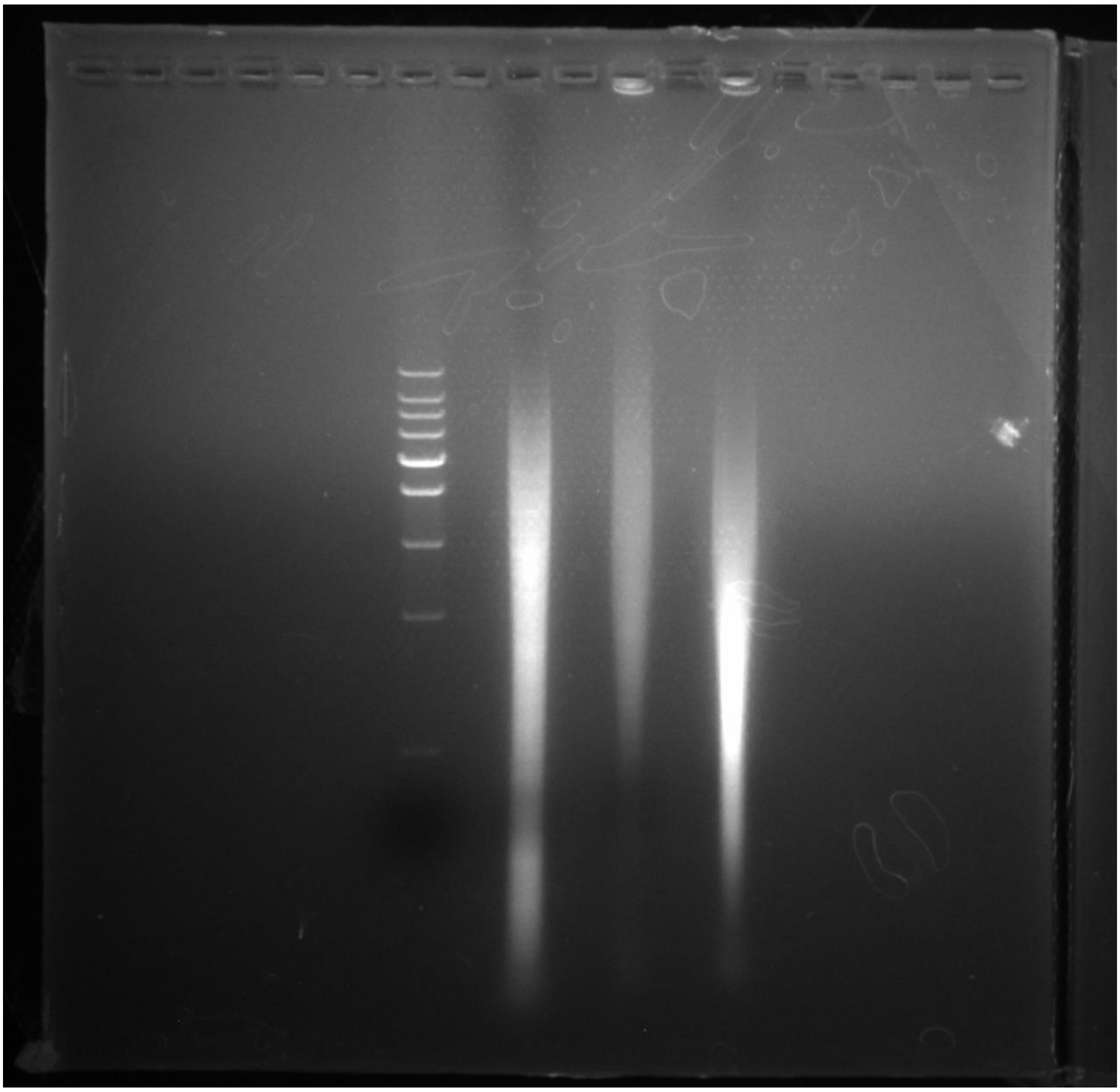

Supplementary Fig. S4. Full gel image of *AoFIG2* for Southern blot. Left to right: 1Kb DNA marker; WT; *ΔAoFIG_2*_S59; *ΔAoFIG_2*_S71.

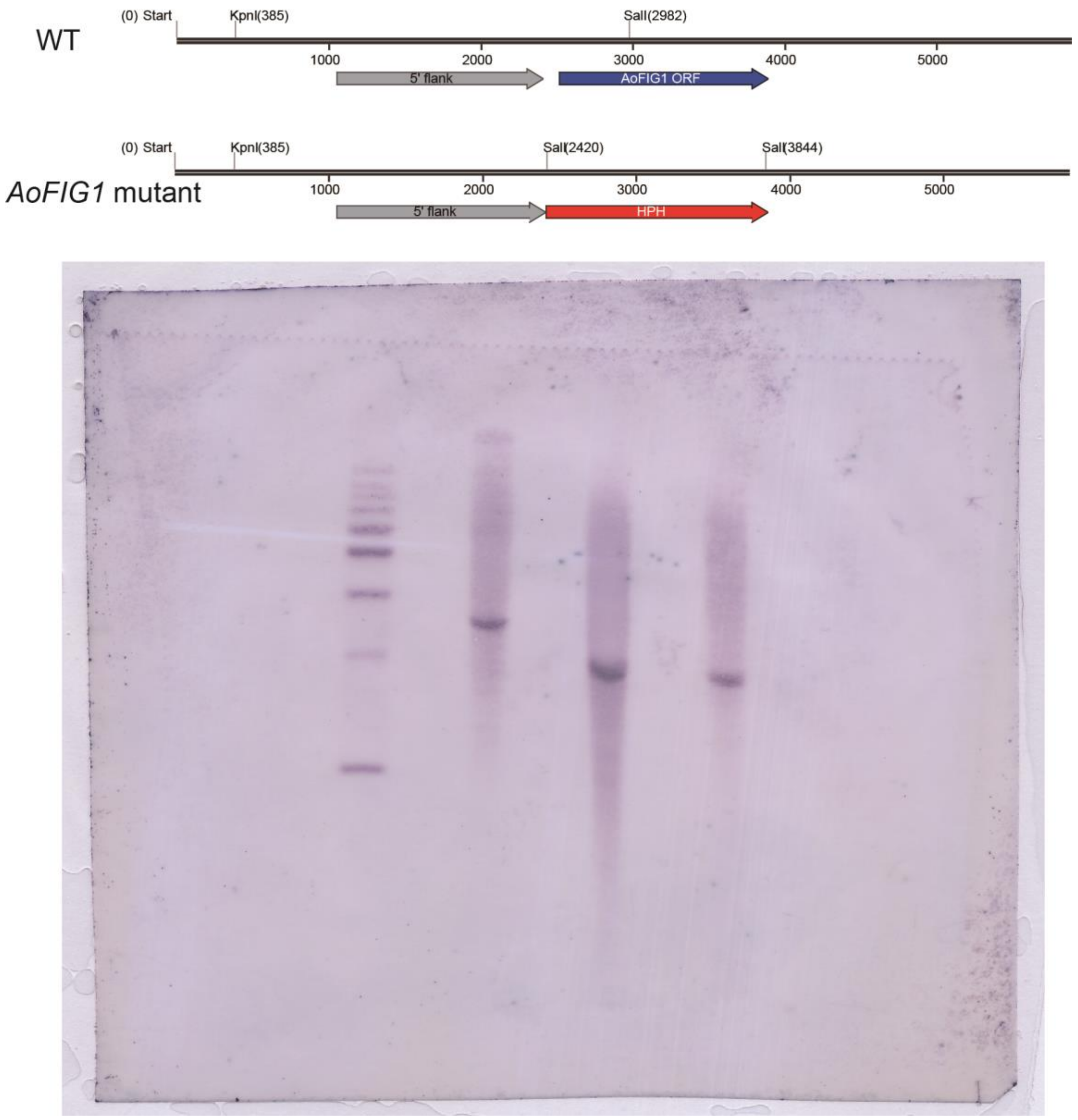

Supplementary Fig. S5. Southern blot strategy and full membrane image of*AoFIG_1*.

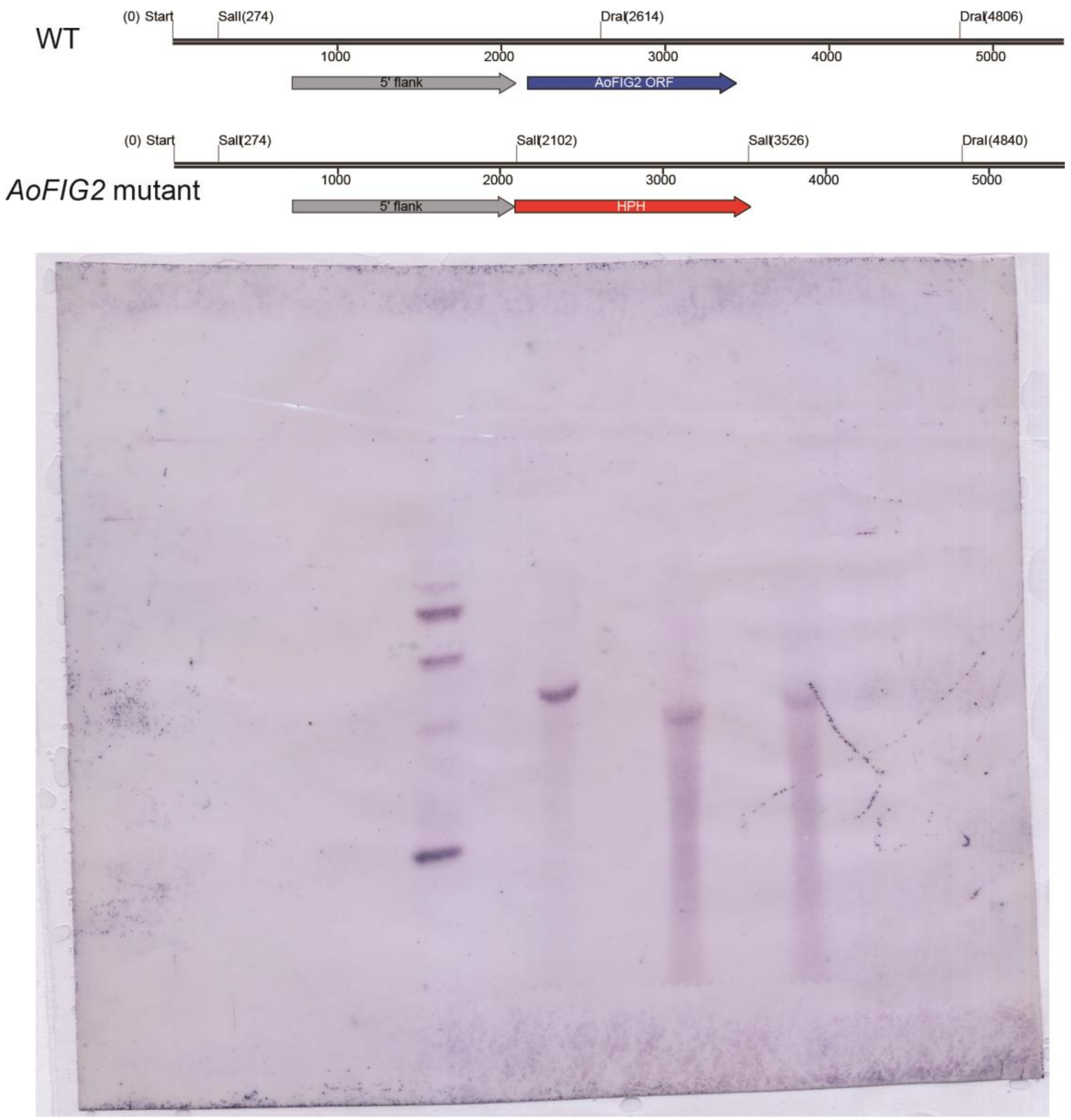

Supplementary Fig. S6. Southern blot strategy and full membrane image of *AoFIG_2*.

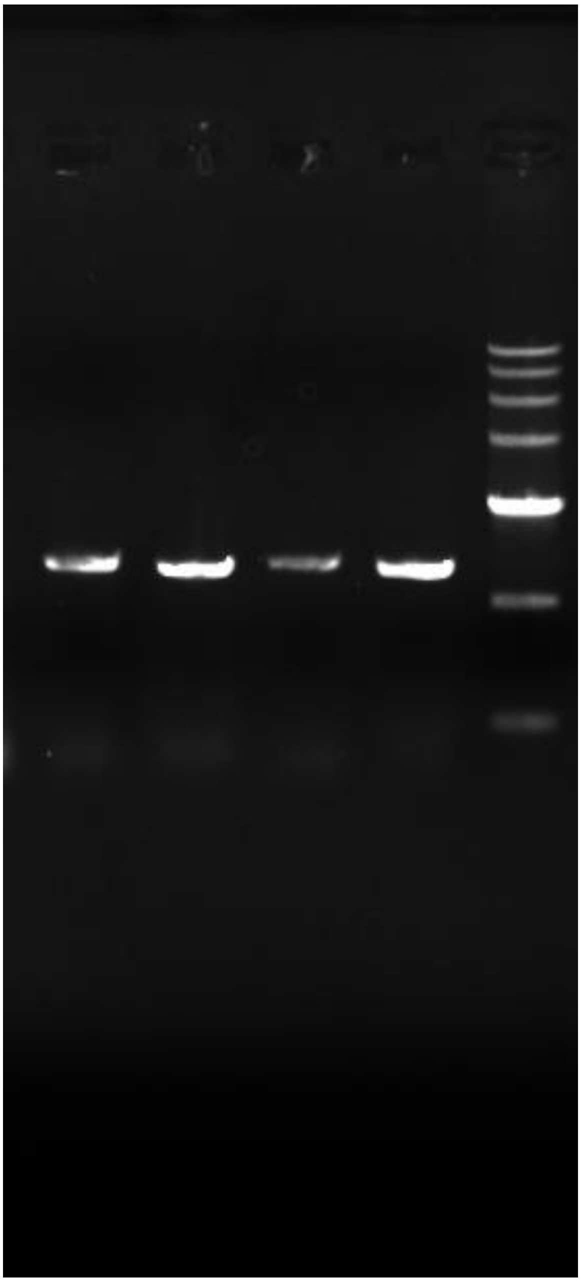

Supplementary Fig. S7. The 5’ and 3’ flanking sequences. Left to right: *AoFIG_1* 5’ flanking sequences; *AoFIG_1* 3’ flanking sequences; *AoFIG_2* 5’ flanking sequences; *AoFIG_2* 3’ flanking sequences; D15000 DNA marker.

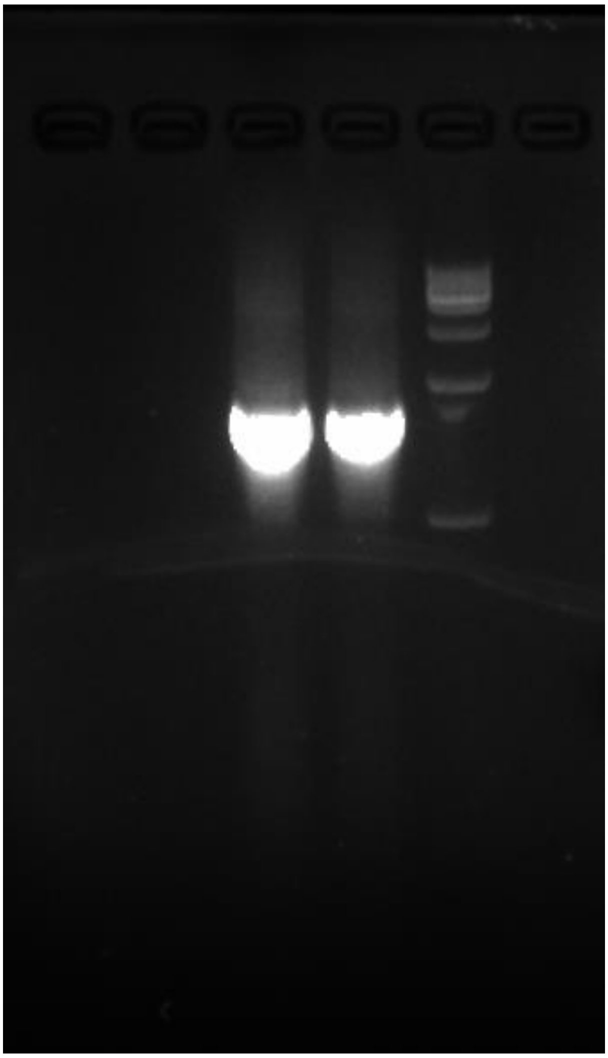

Supplementary Fig. S8. Hygromycin resistance gene. Marker = 1 Kb DNA marker.

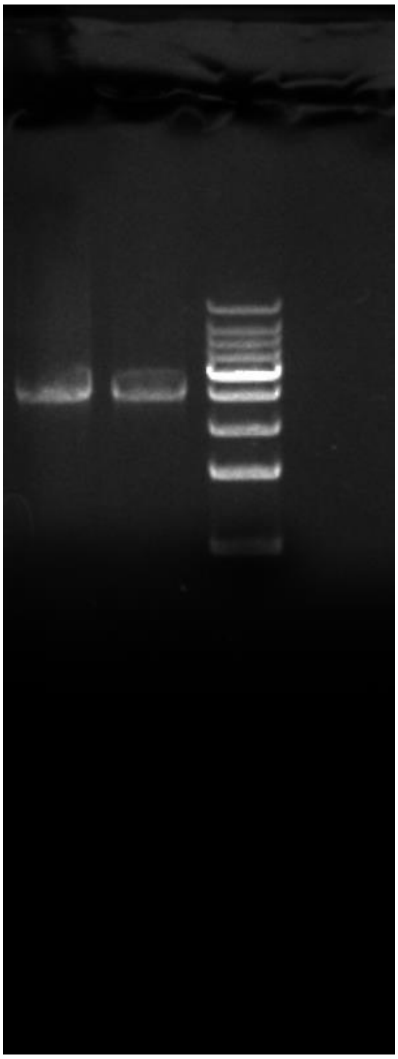

Supplementary Fig. S9. Knock-out cassette. *AoFIG_1* Knock-out cassette (left) and *AoFIG_2* knock-out cassette (right); marker = 1 Kb DNA marker.

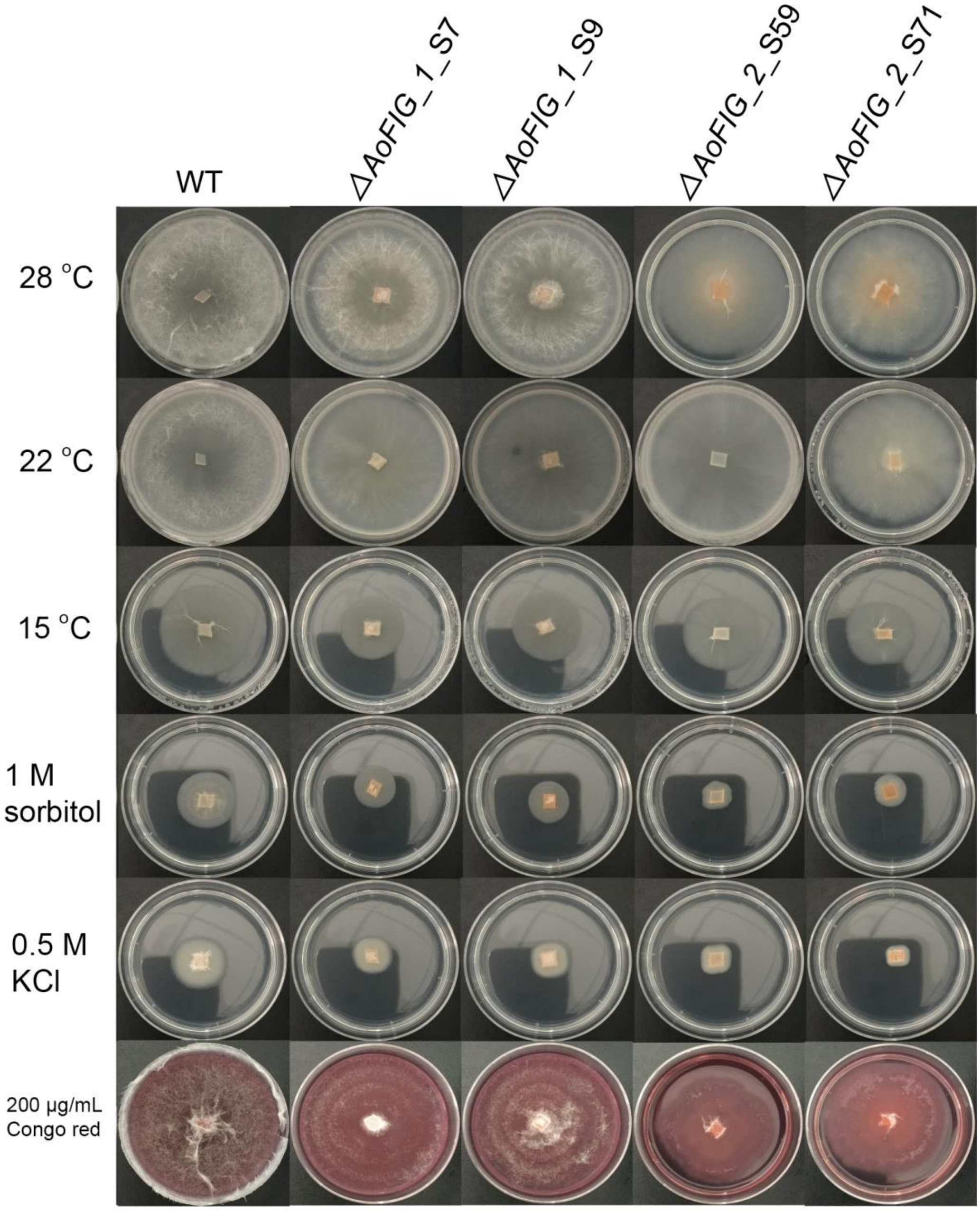

Supplementary Fig. S10. Phenotypes of *A. oligospora* wild type strain (WT), disruption mutants of *ΔAoFIG_1* and *ΔAoFIG_2* to abiotic stresses

Supplementary dataset 1. DNA sequences of AoFIG_1 (ORF was red highlight) and the up- and down-stream (green highlights were the restriction endonucleases used for Southern blot).

GGTTAAGAGGTATATAAGAAGTATGATAATAACATGCGCATCTAGCTGCAGCTCATTTCT
CGCGATCGGTGTAACCTTGAATCTAGGCGCTTCTGATGTGGATCAATGATAGATACCTA
GGTAGATTTGCGAACTAATGTTTGCATACGCAGCGACGTTCAAGAAGTAGTTGAGCGG
ACTTGGGTTAATATATCGCGTCGAGACATTAATCCAAATAGAGCATTGGTATGTTGCCCA
AGCCTAGCGCTGTGCTAGGGTGTTGCAACACAATCGGGGAGGAAAACTTTGTGGTATA
TTAAGTAAGTATGTGGCGTCTGTGATCCGATGTCGGCCAGCAAGTACCTTCCTGGTGCT
TACCTACCTACTTAAGTACCTACTTCGGTACCTCTTGGTACTTACATGCCTAATGCGTGT
ACAGGTCCCGGCTAACTAGCGCCATTTCCTCGATTCGCGAATTTTATTTTACCTTCATGC
AAACTCGCGTGCGCAACCTAATGTAATATTTAATATCATATCAGACTGCCGATGCACATG
ACTACTGACATCCGACTTAATTACATAAGTCTCGAGTTATTTAGCCCATATAGTATACCAT
ATACCATACCATACCTAAATACTTACATACCCACCTGAACCCGCACCCGCTTTTACCGAC
CCTCGTCTATACCTTCGTCTAAGATGCCGAGATCCCTTCGCAAAATACAGTCTCAGATTA
AGAAGAAGCGGGGAGCATCATCCACAGCTCTCGTTGAAGACAGTCGTGACGCCCAAC
GACTACGCAGAGGGATGTTGAGGCACGAAAAACTGGTGCGGTCGGAAGCTCTTAGAC
AAAAAGCACTTCTACCAAAAGGTAAGGCCGGTCAACTTACGGCTTGCTCACTTGCCTT
AACGCCATAATTCCAGCTCAAAGTCTTTAAGCTCCGGTGCTTCCACAAAAAATCCCAG
TTGACAGCTCTATCTAGTAACTCGATATCGACATTTTCAGGAACTAGCGACTGTCGAAC
AATTTTCAGTAGCGGATATCCTTCAGTACATCGAAAGGTACGCCTAGCGTTGCGTATATA
TTGGCTGAATAATCGCAAGCTATATCGTTATTCATGGAACCCCAATTAATAAAAATATAC
GCTAGTTTTCTTTCCCGTCTGGACGAGGAAATCAAGGTATTTTCAGGGGAGCGAAGGC
CCGGCCGACCGCGATCATCGCAGGAAGACCGATTGCGAAACTCTCTTGAGAAAGAGA
TGGCAGAGTTTCACGTTGGATACGGTTGGTTTCAAACCCCTTCCTTGGTTTGCCGTAGC
ATATATAGCATTAAATCGCTCATCTAGACGTCGGGGTTGCTAAACAAACTGTTGTATGGA
TTTAATAGAAGTACCTGATCTCTTTGATCCGGAGACCCGGAGACTCATGTCGGGTTGGA
AAGGGGATGCGCAAGGGTTGGCACAAATGAGGACGATAAGGATCACCAAGGAAGGGA
AGATACTTCCGATAGCGTGATGGCGAGCCTGCATCCCAATGCACCCGTTGGATGTCAAA
TTTTCGATATTGAGTTGATGTCGATGGCGGTATGCAATAGTAATACCACAGCGAGGTAG
CTAGCTATGATGCTAGAGGATGTTATAATCTCGTAAAAAAGAATCGACCCTACTACCTAT
CTCCCGCGGAGTTTTATTTTTTTTAGATATAGATATCCATATACCTACCCCGATAATTAAC
CTTAAGTAGTATGGTATTATATTACCACTTTATGGCATATTATATATCTTAGCATTAGTACTT
ACTTAAGTACTTAAGTAGGTAGCTAGTCATGTAAGCTTATAATTAAGTTACTTAGTACTT
GTAAGTATTGAGCTTTGTAGGCTGTTTGTGTATACACCTCTCGACTCTAGATGAATACCT
ACTTAGTACGTTAAGAAGTAGGTAGATAAAGGTAGGTACTGTACTATACCGAAGTCTCA
ACATACCTCTGTACCTCTGCTGCATGCTGGGTGAGGAAGTATTTAAGCACGGAATTCTG
AATCACGTGACAAATACATGGAGGCTTCCGCCAAGATTGATTGTGGCCATGTCAACTTG
CGTTGCCGTGCCAACTCATCAGCCCCACGATCCCAGCTACGCAACACCACTGTTTACTC
TAATATCAATCAAACCGTTCAAGCTTTAAACCCCATGTTTCTTCATTGAAGTGGACAATA
ATCCACCTTTGGTACATCCTCTTTCAGTTGGCTCAACTAACCTCCGAAATACGAAATTC
AACAGTTGTTTCCCACGAAGTTACGCCATTTCTCAGCTGTCGCAGTCGTTCGTTAGAAT
CGTCTCAAATCCTTTTGGGCTGAGGTCGGAATTGTGTTAAAACATTCGATACCGCTGAC

GCTAGCGCTCCTGGGGCCTCATCACGAACTGGCAAATAACGACACTTCTGCGCATCAA
CATTGGAGCAAGTGTAGTCTTATTGTGCTGGAAGGCTTTATTTACTACCCGTAAAATCG
GCAGTGAACCTTCGAGATATCTTAGTAGATATGCCTTTAATAGAAGTTCGCCGAATAATC
CGGCTACGGCTGACGTTCGAAAGTATGTTGAAAGTTGGTCGGTGATCGCACTGGAAAC
AAATCAATTGCTAACATCTGTGTTTCCAGGGCTGGTTCCCCTACTTGGCTACCATGTATG
TGTGGAACACCCTTCACCAAAATGTCGAATCTGAAAAAACACCCAAATCGGGCTTTAT
ACTCTGGCAATCTTTTCCACAGCGACTAACATACACAACCTAATTCCAGCATATTTTGAT
GATATTCATTATTCTTGCAATCATTTTACTATGTCAGTCGGCCCTAGGAAACGTTTGCTTG
TAATATGGAAGCAGAAATCTAACAACTAAAATAGCACTATTACTTGCAGGATGCTCATC
ATCGAGCCCTCTGATACCTTCAATATTTCTGATAAATCTTTTCTACAATCATTATGCCCCG
GTATATTCGACAGCTCAGGTCGACCCCGAAGCAGCTCGGGCGATAGGAAACATAGTTG
CAAACGCCATGCTTGAAGCTCGAGTCGGGTTAGTACCCGACCTCCTCTACGACACCAA
CTGTGTTGCATATTTCCCTCTCTAGTTCTATACCTTATAACTAATATAAAACGCCTCTATA
GATATTTCGGAATCTGTATCAACCCGGATGGTGGTTCTTGGCTTTGTAGTAACAATGCGT
CCTCCCTTGCAGCCCAAGTAAGTGTTGACCAAGACCCCCTTAACCTCATTTGGGTGTC
AGATACTTTCCGGGATAGTATCGTCTTCCCGTATCTGCTGTAAGTCCCCCTATACAATATA
TGAACCGTTCATATGGCTCACGTCTCCCAATAGTATCGTGGCAATACTCCTTGCTACGAT
TGTTTTGGTTATGCTCGCAACATTTCCAACCTATAATAGTGAAGAAGAGAGAATGCCAA
GCCGATTCGTTTCGCAAATCGCACTGGCAATCATCTTCATATCTGCGATATTTGTCCTAG
TTTCTGTCTTATGGCAGGTTCGTATTTCACGAGAGAATTCCTGTATATGCCAAGCTAATA
TCCTATTTAACTAGCATACTGCATCGATTGCTGCAGCCACAATTGCAGAAAATCTTGGA
AATGGGTCGATACAGACTGGCGTTGGTGTAAGATTTCCAAGCTAATACAGTCACTTGTT
GGGCGTTTTAAAGCTGACGAGGGAAAATACACTAGGTCACAGCACTTGTTCTGGGTTG
GTTCGGCTTTGCTTTACTTATTCTAGTGACAATTGGATTGCTAGTAATGTAAGATTTCTCA
TATAGTTCACGAATTTGGGCACAGTTAATACTAAAATTGACGGTGATTCAGGATACTCTC
AAGGAACCTGGTTGACAAACAAATAGTCGATGACGATGAATAGAGTTCCAAAACGGC
CTGCGAGTTTTACAGCTCGCCTCCAGGGGTATAAGTCTTATTCATTGGAGGAGTTACTT
GCTACTTGCAAATTGATTATATGTGGGTGGGTAACAGGCATTCTGGCGCTCTGGGTTATT
TTGTGTTTTATAAATGATCATTTAATGCATTCATATAGGGCTGTCGTGGGATAAGAAGTTA
AACCCTTTATCCCTTCGTTTCTATTTTATATTGTTTTCTATTATATTTACTTTTCCTTTTTTG
AGACCTGTGGTGGGGTTATGAATCTGATGTTAAATATCTCCTTTGTTTTTCATACAAGCT
GTTAACGCATGCAGACTCTTTTCGCTTGTGGTCAATTTAATTGGGATTCGAATTTCTTAG
TCTTTGGACTTTGTACATACAATGTACATACGTATTAGATGTACTCTGCTACATTTGAAAT
TTCCACCTACAACATTTTATATTCACATGATCATGATCTTAGCCTTTGGAAGCTTTGATCC
TTAATACACGTATGCACATTGAACGGATGGGGAGGCTTGGTATAGAAGGTTTTTAAAGA
TCCATGTACTGGGGAGATTATCTCTTCTATTTGCTTGATAGTGACTGTATTATTACCTTGT
TTTCTAGAAAATCTATACTATCTTTATAAACTAACTATCCTGAGGCCCTAACTTACTCGA
AATCCTTTGATTGAACGAAGGTTGTCCGTGGCCGACCAATGGTCTCCACTTTGAAACC
GATCTCTGAAACCTTCTTTGCGTCTAGTATAAGCCGGCCATCGAAAATGAAAGCAGGTT
TCTTCATACTCGCGTATATTCGCTCGTAGTCGAGAACTGTTTCCCTGAAGTCGTTCCACT
CGGTGCAGATTACGATGGCATCTGCGCCAGCGGCAGCTTCATAGGGGTTTGCAGCAAT
CGATACTTGCTTCTTAATGACTTCTGTCTCGTCCACCACGCCTGGCTCTGTAAGATCCAT
CCAGACCTGTGACTCTTCAACCTTCGGATCATATATCGAGATGTATGCCTTTTCTTCTCG
AAAGTAATTGACCAGTGTGATAGCGGGAGATTCGCGAGTGTCGCCTGTATCCTTTTTAA

ACGCAAACCCGAAAACGGCGATCTTTTTTCCAGTCAGTGTGTTAAAAAGGCAAGATAT
CACACGATTAGTAAACCTGCGTTTCTGGTATTCGTTCATCTCAACGACCTATACAAGGAT
TAGAACAAATATTCACCTCTTCTACCCCACAGGGATGGGGGGTAGACTTCACGCTTACC
TGCTTCCAGTAAGTAGCGACTTCCGGTAGGTGCAAGCTTTCGCTAAGGTAGACCAAGT
TCAATATATCCTTTTGGAAGCAGCTCCCGCCGAATCCCACACTAGCCTTCAAAAATTTG
GGTCCGATCCTAGTATCCAGTCCACAAGCATAGCTGACTTCATCAATGTCTGCGCCAGT
TGCTTCACATATAGCACTGAGAGCGTTGATACTTGAGATGCGTTGGGCGAGAAGGGCA
TTTGCAGCCAATTTTGAAAGCTCTGAAGACCAGAGGTTCATGGTGATGATTTGGTCTCG
GGCAACCCATCCTGCATAAACATCCGCTAGGGATGCAGCAGCAGAACGGCCTCTTTCC
GTATCCAACGATCCAATCAAAACACGGTCCGGATAGAATAGATCTGATATAGCAGTTCC
TTCGGCAAGAAACTCCGGGTTTGACAGGATGTCAAAGTGAATATCCGGCTTCGCATTT
GCTTCGAGCTGGAAACCCCAGGTGGGGAGGGTGTCAGTGTTTGTCGAGAAAGATCCT
AGGTGGGGTAGAAATAGATTGGGAGTGCTTACGATGTATCTCATGGACTGGGCAGTTCT
GCATGGAACGGTGCTCTTTTCGACGACGATCTTGTCGGATTTCGCAACTTCGGCGATTT
TCCGCGTGGCACTCTCGACATAGCCCAAGTCCGCTGCAAACCCAGCGCCTATTCCCTT
GATCTTTGT

Supplementary dataset 2. DNA sequences of AoFIG_2 (ORF was red highlight) and the up- and down-stream (green highlights were the restriction endonucleases used for Southern blot).

CAACCCAATCTCCACATAACTCGCATGTTTTGTTTACCATGACCGACACAACGACATCA
TATTTGATCCTTTAGCGACCGATCATTTTCTGCATGGGCCGGTGTTATGCTTTGTCTTTTG
ATTTTATTTTTTTCTTCTCGTTTTACCCCGGACCAACTACAAATGATTTCGACTATCTTTA
CCCTCTTGTCACGATTCGTCCTGATACATCAGCCATGTACCCTGGCTCCAACTCCGAAC
TATTCTCGTCGACTAAAGAAAGAAATAAAAATGGAGAAAAACCAACTGTTTGGCCATC
TGCCCCATATTGCAATGTCTGTCTACACTGTTTACTCTTTATCATACTCGTACGATTGCAT
TGCCTTGGAGTTTTCAACGGACTTGGTGTTTTCTCGGACGTATGCTTGCCTTAATGTTT
GTTTCAATCAATTATTTTAATACTTTTTTGTTACATAAATTATAATAGCCCGCACGATACA
GTTTTTAGCCAACTGTATGGTTTGCGGTGATTTTTTATATCAATGATAGCAAAATGTAATC
TGATCAACCCCGAGGCTTGTGCAAAAATCTAGATACGACTAGTAGAATGACGGCTACA
GCCACCAATCTGCTGCATGGTCTGGCATATTTGATGGCTTATCTGGTTATGGATAGGGAA
GGAATGGAACTAGCTGTCATCGGGACTTAATCCCCGGGACGTCCGGAACGATACATCG
AACTTGAATTGTTACACAACGGACAATGTGGTGATTTACGACGTCGATGTGTTGTCGTC
CCAAGTTGGGAGTGGTGACAGGGGTATTCTGGCAGCTGATTGGCCCTACTGTTTAAGC
GCAGACTATGAAACATCTCCAAGAGATAGCGCCAACCAATACACTCCCTCGCACCGTT
TCTTCCCCCGGATGATGTCCTAGCAAATCATCATACTCAGAGTCCAGCTCACCACCACG
AGAGGCCCGATCCAAAAATAATGCGGATATGGAAAGAGCCGCATGGTTTATCTACTAGA
CCGACCAGGACCCAAGTTCTGGTTGTCGAAACGCGTATCAATGACTGGATCTTGGGCT
ACCACCAGCACGCGCCCGCCACTGAACCCTAGCTTCTTTTTTCCTCTTATTGAAATGCC
GATTAAATAGGTTGGGTATTAAACTCCCCGGCAGCGAAGAGACTGGATTTATCGTAATC
TCGACTACTGGTGCCTGGGCTTTGTACTTTCCAATAATAAGTACACTTATTGGCGGGTAC
CAGAGGAATAAGCTCAAGTACCTATTCTTAGGTTGACAAGCTTACGAACTCACGGATTC
GACATGCCACTCGCGCCTGGGCTAGGGCAAAGTTATGGTAATTAATTATACGGTTACTAT
GAATACACACATGCTGATCGGAAAAAATTCTGAAGCGTTAACAATACCTACCTGGCGA
CTTTGATACGGTTCGCATTCCGAAGGGTGGGGCATGCCATACAGCACGCCTCGCTTGAT
TCCAGGAACAGGTGGTGGCCCTCTCTCACTGCGCGAGTCGCGCATCTGTCGAAGGATG
CATGGAAAGCTTGTCATTGGAAGCTATAGGGCGACAGCTCAACTTAATCCTAGCAGCG
GGTTTTCAGGGCAGCTGTCTTCGTTGGTCATTTGAGATATAAGTTCGAAGTGTCGCCCC
CCCTCAGTGGTTCTTCTTATTCTCTCCCTCATCCAGTTCAGTCTGATACGATTTTCTTTAC
TGTTTCGTAGCCTTTTCCTTTCCTTTCTTTCCTATTCTTTTTTTTTTAATTTTCATTCTTTT
CAATCAAACAGCTAGCTGTTGAAAGTGCAGAGCACTTGGTTTCTTATCAGATCTCATAT
AACTATAACTATCTTTTTTTTCTTTTTCTTTACTACTTTCTTAGTATTTTTTCTATCTCATTT
CAAAAATTAAGATCAAGCCTTTTTGATATCAGAGAGCAAGTATATACTTCTATACATATT
AGAAAGAGAGAAAAGAGAGAACGCAATTGGCTTCTAGAAAGAATTTTTTGTTTGTTTG
AAGAATTATATCAGAAGGAAGGAAGGCTCCAGACCTCGTCTCTCCTAGACCAGCCATC
AACACCAACCATCACCAACAATCATCAACCTCACCAACCTCACCAACATTGATAAAAC
CTCGAAATGATTAGCATTGCCAAGTTCGCCAAAGCGGCGCCATGCACAAGTATGTAGTA
GACCATCTAGGAAAAGATTCATGCAGAGTTACTAACCAGCGGTTTGCATATAGAACCAC
TCCTTGGTTTCGCCTCTCTAGCTCTCATGATCGCGTTCATCTTCTCCACTGTCATCGTCA
CAGCTGGAAAACAAGAGAGTAGCAAGGGATTATACATCGTCTCTTTCGAGTATGATGTC
CCAGCTAGCAATAACAGACTCGTCAGGCGTCAGCAGGATGATGATGAAACCACAACCA

TTGTTCAGACTCGTTCCGTTGCTACGATCTCTCAAACGAGAACCGTTGCACCAGAACC
AACTGAGGATCCAGCTCCAGAAGAAAAAAAGGAAACCACTACTCCTGCTTCTTCCCCC
ACGCCAACGCCTACTCCAAACCTCAAGCTTCAAGAGAAATTTAAACAAGCAGTCGAAT
CCCTCATTGAAGACGACAAAATGAATGCCACCATCCAGTTCAACCACGCTCGTGTCAG
CTACAGCGGAATCTGTGTCGAAGTTACTTCTATGGAAGGAATCAAGGGTGCCCAATGG
AGATGTGGACCTGTCAATACCACTGAAGTCCTCGGAGCCACCGCTGGCGGCGATCCAT
TCGATCTCATTGGCGTTGCCGCATTCTTCAAGGATAAGATTGCATTCTCCCTTCCATGGT
GGGTCGCAACTGTCTGCTTGGGTATCGCCATGTTGTGCCAAATGTTGTTGATGCTTCCA
CTCCTTCCTATCCCACCTGTCGTCCAGAAGGTCACCGCCGTTCTCGCCCTCCTTGGATG
TACCGCACTTCTTGGAGGCTTGGTCCTACAGCATGTCGCTGCTAACACCGTTGCGTCGC
TGTCTCTCAAGTTGACCATGGGAACCGTCAACGCTCACGTCGGAAGAATGAACCAGG
CTCTCGGATGGACTGGTTTTGCTTTGTCCCTGCTTGCAAATATCGGAATCTGGGTTATCG
TGGCCGCTGAAATGGCAATTGAAAAGGGAGAGCAAATGATGAACCATGCTACCGACG
CCGCTATCAACAAGGTTGAATCAAAACTTCCATATGGCAACCCCGGACAGAACAGGAG
CTTCAGCGGATCGAGCACCACTAGTAGCGGCCTCGGAAGGTCGCTCAGGGATAACGGT
CCAGATGTTCTTCGAGGATTGGCTAAGGCCAAGACAAGGAGCGAGGCATTGAGTGCTG
TCGCTGGTGGGTTTAGAAATGAGAAGACTCATCAGCCCCATAACATGGTCTAGATGGG
GAAAAGTTTAACAAAAGAATCAAAAGATACCCCCGTCAAAGTTGATTATGTGGGAAGA
GTCTATTAGCTATATGATTCAAGCATCCTGGATGCCCTTTCTTCATTCCTTATGTCATTCG
TTCATTCGTTCATTCATTTTACTACAAAGCATTGCATTGCGTGGACTGGGTCTCTGTCTC
TCTCTATATATTTATTTGCTTCCTTTCTATCCATATCAATAAAGTCTTTCCTAATTCCGAGA
ATTCCGACGGTCCTTTTATGTTCCTATGAGTCTCCAAAAACCTTGAATTCAAATGAAAA
AAAGTATCTTTGTATTTGAGCTTCAAGTGAGATGTCAAATGTTAAAAGCTTCTCTTCTCC
CGCTAGCTGTTGGTCGTAAAGTGATTAAGAAAGAAATATATCGGGAACTTCAGCTCAA
AGGATCATTAACCCGACTAGTAAACCATGTCCCCTCTGACGTCGTTGATATACGCCTAA
GTGCCTCTTGAACATTAATATTACCAGCTAAACTAGGATACTAGCTTTTCGTATTGATCG
GCAGTGTGTTGTGGAGTTGGATATAAGAATAAATATCTGGAGCCCACACACATAACCCT
GGGCTTCCACCCTAGCGATACAAAAAAAAAATTATTTCTGCCCCTCCCCAAGACAGATC
AGGGAAGGACAAAAGTACATCGAATACAGAGGATTTACTACTAAGGAGTTATGGTATA
ACCCAAATCTGGGAATTGATCATGAGCACGGGTCTCGCCGGTCCACTTGATTCGCCCAT
GAGAGAGGCGGAGTCTGACCTCTGATCCAATATGGGGTTCGGATAGAAGACACGGTAT
ACCGGATGCCTCCCACACGGCTTGCCGGGGTGCCGACGATCAGGAATTTGCCGACGGC
AAACCATTTTCAATCGGATCCGGTGGATCAAAGGATGCTAACTATGCAGCAGATGCATT
ACCGGAGAAACACTGCTCGTGTTCCTCAGCATATCATCTTTTGGATGTGCAGTTGAATT
GAAAGTCGTGGCATGAGAGGATAGGACAGTCCTTTGTGTAATTGTGAACCATCGACAC
TGAGTTTTTTATGGGAGCAATCCAGAAGCTGTTACACAGGCGGCTTTCCGGCGCTGTG
ACCTAGCAGCGCACCTGCCGGTGCGACAACCATCCATTCGGCAGGAGGCGTGGCCTCG
GTCCAGGCTGGTTAAAAAAACCTTTCCAATGGCCCCGAGTCTTGGCATGTCAAGATGT
TGAGCTGTTGGGAAGAACCAGGGACGAAAACTGGCAATGATCAAGCTAGTCCTATGA
CAGGAAATTCACTGCCTGGTAGAAAACGGTAGACCTCTTGATCAAGGCTAGTCGAAGC
TTTTTTAAACTTGGAGACATGCATCGGTTTTAGTCTAGCTGCTTCGTCTACCCTTGTCAG
CGTGGGATGTCATTATCAAAAACATATGTATCGATAGGGTTATCAATATACCTGATGCTCT
AAGAGAAGAACCCCCAGTGCTCATACCATCTGTATATTTATATCCGTTGAACCAATGCAT
TTCGTTTGCAGCGATTGCGGGATCGTGTGAGCATCCATACATCATGCATGACTGAAGGT

TAAAACAAGCGAATCGGTTGGCTCTGTTGGCTATTCCCACGGCAAACCATAGTCCCTCG
AGGGCATGCCACCTTAGAGAGCACCTTGCATTAAAAATGTTGGATAATCACGTTTGCGT
AACAAGGGAGGCCGTTTTCTGGCTTGTGAAAATTTTCGTGAGGGTGGCCCTGGGATAA
TTCTTACGCCCACTTGGCAAGTGAATCGAAAGCTGTCGAAATCCATGAAAGGGACAGA
AGGGAGAAAGAAAGAAAGAAACCGAAACGGCGTGGGGAGACAGAAGCCTAAAAAC
CGGAGCTAAATAAAACTGCCAGCCGCCTTTCTACTTATGCTCACACGACCCTCCCCCAT
GGTAGCCGACAATCTGTAGAAGGCCCTTCTCCTCTAGGATATTTTTTCAACCTACATTTG
CCCTTGATTTTCAACCTCAAGGGCTGTAGAACGACTGTGGGATTTCCATAACCCAAGTT
TCCGAGTGTCGATACCCGTATTTTCTCCCTGCTGGCTTGAAGCATAGGAACTCGAACCA
TGGCGACAGGCCACACCTCTGCTGACCTGCCTGGCACGGCCAACAGCACACCCCAGG
TGCGGGAACGGACGAAATCCTCGAAGAAACAGCAGAAACTATCGCCCAAGCTTACGA
CGGAGACTTTGAATGATGGTGATTTGAAGCCGAGAAGTGAGATTGAGAAGGAAGCTA
AGACGTATGGCCGGACTCCCGATGGAACTGGTAGGTCACACAGCTCAGCAGCATCCCG
TCAATCACAAGCCGATTGATCTATGGACTCGACCAATTGGGAAATGTGAAGAATGCAA
CAGCTGACAACGCATCTCATCTCGACAGTCTTTGTCGTCCCCCAAACTCATGATATGGT
CTCTACCCTCCTCTCGCCCAAGGAACCAAAGAATCTCTCTGATCTCCTCGTCCTGGGTG
TTTTGGCGTTCCACATAAGCCTATTGTTCATCCTCCCGCAATGGTCCCTAAAACCAGTGT
TTATGTTTACGTTTCTCTTTTGGCGGGCCGGATACAATCTTGGAATCGGCCTACTCCTCC
AGTCTCAGAGCAAATATAAACAGCTCACACACTGGGCTGCCAAATACAAGCTATTTGA
CAGAGAAGCTCACCCGGTTCTTTACGACTTTATCAAACGTGAAATCTCAACGAAGATA
CCCTCCGAAGACTATGACTTTGATAAAGCACCACTCGAATACAACACTTGGATGGTGTT
CCGTAGGCTTGTGGATCTAATCCTCATGTGCGATTTCGTGAACTATGTGCTATTCGCCTA
TGTTGCCACTGAAGTACCTACCAGCCATAAGATGTGGTTGCATGTCTGCAGATGGGTCG
CAGGAGTGGT
